# Building epidemiological capacity to strengthen health systems: evaluating the advanced (extended) field epidemiology training program of Papua New Guinea

**DOI:** 10.3389/fpubh.2026.1777107

**Published:** 2026-03-03

**Authors:** James A. Flint, Jennifer White, David N. Durrheim, Barry Ropa, Alois Pukieni, Callum Thirkell, Miriel Boas, Elaine Hevoho, Martyn D. Kirk, Tambri Housen

**Affiliations:** 1 School of Medicine and Public Health, College of Health, Medicine and Wellbeing, The University of Newcastle, Newcastle, NSW, Australia; 2 Surveillance, Emergency Response Policy and Coordination Unit, National Department of Health, Port Moresby, Papua New Guinea; 3 Public Health and Clinical Services Coordination Branch, Autonomous Bougainville Government Department of Health, Buka, Papua New Guinea; 4 TB/HIV Global Fund/COVID-19 Project, World Vision Papua New Guinea, Port Moresby, Papua New Guinea; 5 Technical and Advisory Services Division, Animal Health Laboratory Services, National Agriculture Quarantine and Inspection Authority, Port Moresby, Papua New Guinea; 6 National Centre for Epidemiology and Population Health, College of Law, Governance and Policy, Australian National University, Canberra, ACT, Australia

**Keywords:** capacity building, field epidemiology training program, impact evaluation, public health workforce, theory-based evaluation

## Abstract

**Introduction:**

Papua New Guinea (PNG) has implemented a three-tiered Field Epidemiology Training Program (FETP) to strengthen their public health workforce. The first advanced (extended) level FETP (aFETPNG) commenced in 2019. This study evaluates the outputs, outcomes and impacts of aFETPNG.

**Methods:**

Guided by an FETP impact evaluation framework, a mixed-methods evaluation was conducted to assess program effectiveness and identify opportunities for improvement. Data were collected through an online survey of graduates and interviews with graduates and senior-level government managers and executives. Quantitative data were analysed descriptively. Qualitative data were analysed thematically; findings were triangulated and assessed using contribution analysis.

**Results:**

Despite disruption from COVID-19, 17 of 30 (57%) trainees graduated in 2022. Graduates reported high confidence in and frequent application of core field epidemiology competencies. All reported applying field epidemiology skills in their workplace. Graduates strengthened surveillance systems, investigated outbreaks, and implemented a range of public health interventions that contributed to stronger health systems and improved public health programs. Most graduates (82%, *n* = 14) used surveillance data to guide program planning and delivery. Fifty-five outbreaks were investigated (average of 1.8 per graduate per year). Many assumed leadership roles during the pandemic, and 47% received a promotion following graduation. Barriers to applying skills included unsupportive workplaces, heavy workloads, limited resources, security challenges and professional jealousy; enablers included active professional networks, supportive workplaces and communities, and recognition of the field epidemiology skillset. Senior managers confirmed that aFETPNG strengthened health system performance and recommended institutionalising and expanding the program. Graduates recommended more structured mentoring during and after training. Senior managers recommended formal qualification recognition and a pathway toward a master’s degree to support career progression.

**Discussion:**

aFETPNG made a substantive and credible contribution to PNG’s public health system demonstrating progress along the theory of change impact pathway. The program enhanced outbreak response, improved surveillance practice, strengthened public health programs and delivered tangible benefits at community and system levels. These achievements reflect the intentional design of the program and the sustained application of skills by graduates. Key recommendations include strengthening mentoring during and after training, expanding post-graduation professional development, and securing funding for graduate-led projects.

## 1 Introduction

A capable field epidemiology workforce is central to national health security. Countries require health professionals who can analyse surveillance data, rapidly detect and respond to disease outbreaks, and translate evidence into timely public health action. Field Epidemiology Training Programs (FETPs) are a key mechanism for developing this workforce capacity. Since the Epidemic Intelligence Service commenced in the United States over 70 years ago, the FETP model has expanded to more than 150 countries worldwide, substantially increasing national, regional and global preparedness and response capability for public health threats (1).

Papua New Guinea (PNG) was an early adopter of the three-tiered FETP model in the Pacific region. The tiered model includes Frontline (3 months), Intermediate (6–12 months) and Advanced (2 years) levels, providing a pathway for progressively strengthening field epidemiology competencies (2, 3). In 2013, the National Department of Health launched a 9-month intermediate-level FETP to equip trainees with skills to strengthen routine surveillance, conduct outbreak investigations, design operational research projects, and implement evidence-based public health interventions (4).

With funding support from Australia Aid, PNG commenced an extended intermediate FETP, known as the advanced Field Epidemiology Training Program of Papua New Guinea (aFETPNG), in 2019. This two-year program, tailored specifically for the PNG context, sits between the intermediate- and advanced-level FETPs accredited by TEPHINET, the global network of FETPs. Unlike accredited advanced programs, which emphasise analytic epidemiology and advanced statistical methods, aFETPNG was designed to consolidate and expand the practical field competencies developed through the intermediate program. aFETPNG is embedded within the Papua New Guinea’s National Department of Health; it does not provide a university qualification such as a master’s degree. To date, PNG has graduated one cohort of trainees from aFETPNG.

**Figure 1:**
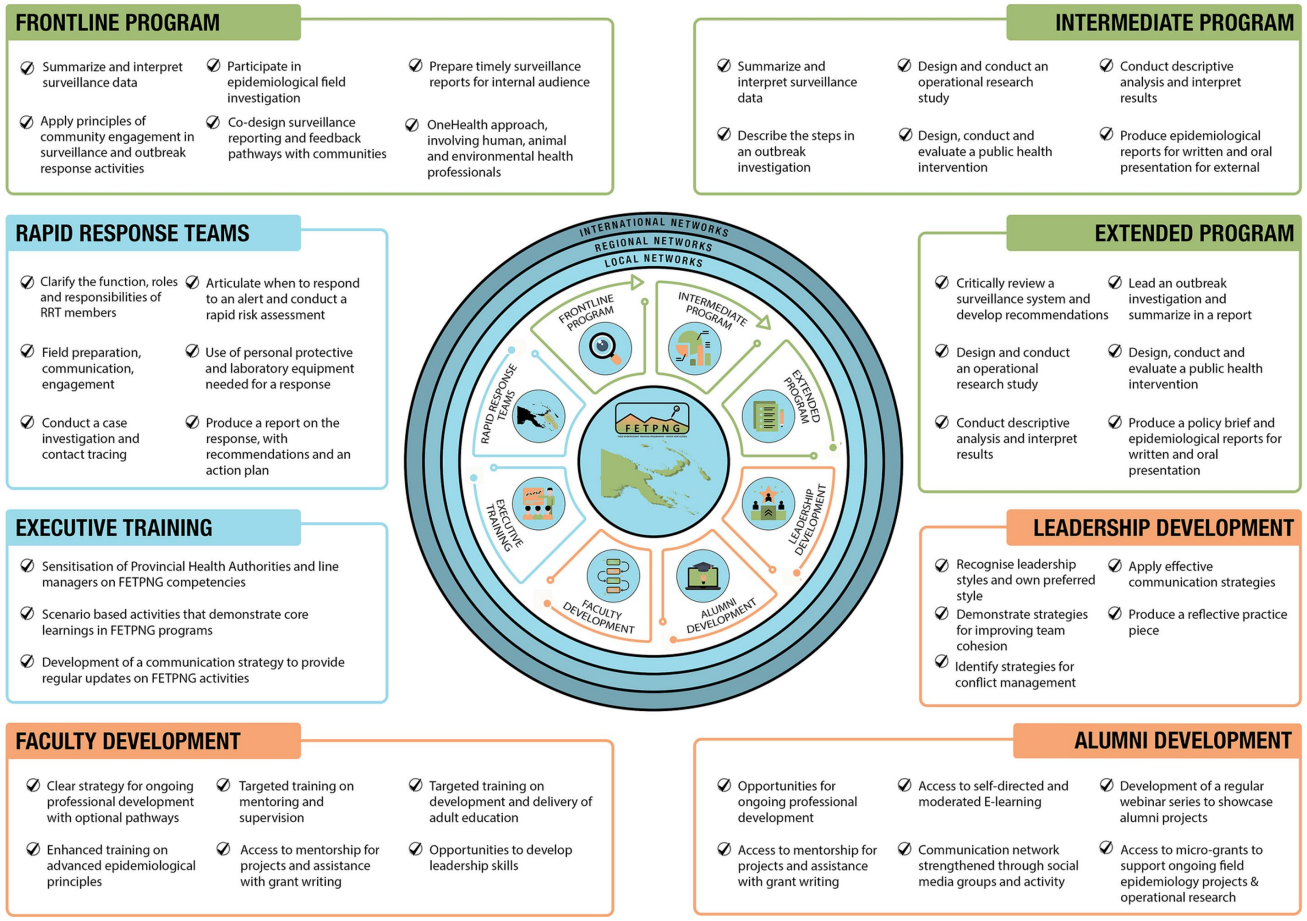
Papua New Guinea’s training programs for strengthening field epidemiology, rapid response, and public health systems, 2025.

More recently, PNG introduced a 3-month One Health FETP called the Frontline Field Epidemiology Training Program of Papua New Guinea (5, 6), completing the three-tiered FETP training approach that has been adopted in a growing number of countries and regions worldwide (2, 7). In addition to these three levels of FETP training, the National Department of Health, supported by the World Health Organization and the University of Newcastle’s Field Epidemiology in Action team, oversees a Rapid Response Team (RRT) training program (8), along with a range of complementary programs to strengthen the sustainability and effectiveness of PNG’s FETP and RRT training programs (Figure 1).

**Figure 2:**
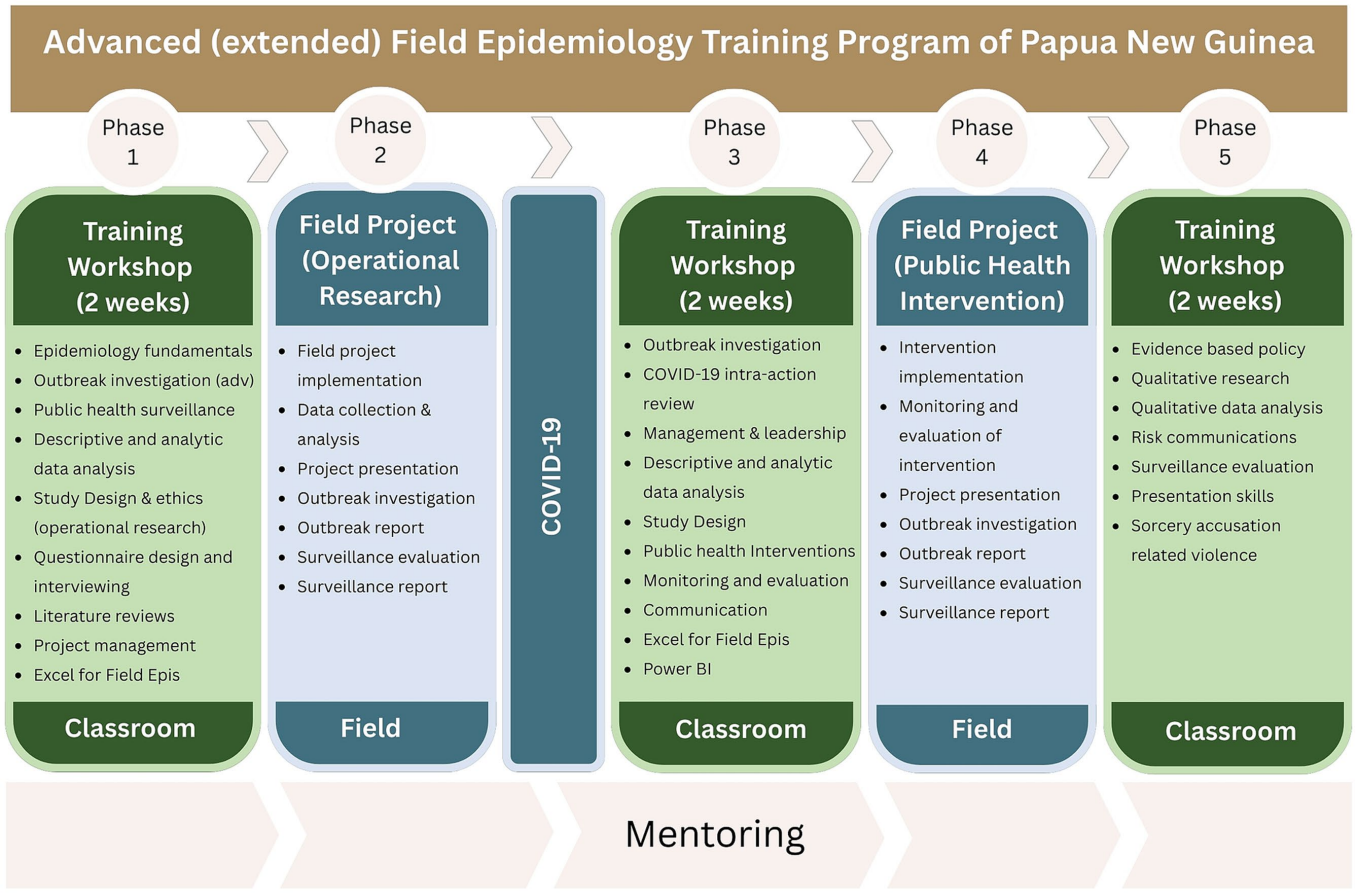
Structure of Papua New Guinea’s advanced (extended) field epidemiology training program, 2019–2022.

aFETPNG was comprised of three classroom workshops, each of two weeks’ duration, and two field phases (Figure 2). The first workshop was delivered in 2019, followed by an initial field phase where trainees applied their epidemiological skills to investigate a priority health problem. Field projects typically involved analysing secondary surveillance or program data to define the problem, and collecting primary data to identify underlying drivers and contributory root causes. The second workshop, originally scheduled for 2020, was postponed due to the COVID-19 pandemic, during which all national and international faculty and trainees were directly involved in the response effort. Remote training was conducted during 2021, and the second face-to-face workshop was held in early 2022. During this workshop, trainees designed public health interventions informed by findings from their initial field projects. These evidence-based interventions were implemented and evaluated during the second field phase. The final workshop was held in late 2022, with a total of 17 (57%) trainees graduating. While aFETPNG was designed as a 2-year program, the interruptions due to COVID-19 resulted in the program taking over 3.5 years to complete.

Prior to commencing aFETPNG, a theory of change workshop was conducted with FETPNG faculty and partners to articulate the program vision and map the intended pathway to impact. A summary diagram produced during the workshop is included in Supplementary Annex A. The program objectives were to: (i) strengthen field epidemiology capacity, including in disease surveillance and response; (ii) develop an active alumni network; and (iii) strengthen health systems across PNG. In addition to the theory of change workshop, a consultative workshop was held with 39 representatives from various departments and organisations in PNG, including the National Department of Health, Provincial Health Authorities, the World Health Organization and the National Agriculture, Quarantine and Inspection Agency (9). The purpose was to identify and prioritise operational research areas for aFETPNG trainees based on perceived public health importance. Four key priority areas were identified: (i) vaccine-preventable diseases and immunisation; (ii) health systems strengthening; (iii) maternal and reproductive health; and (iv) communicable disease control. More than 50 operational research questions were subsequently developed to guide trainees in their field projects (9). Trainees were organised into four teams, each focusing on one priority area. Shortly after the program started, the COVID-19 pandemic prompted aFETPNG to rapidly pivot to supporting the national response and introduce a fifth priority area—COVID-19 (9). Trainees applied their field epidemiology skills to response activities, and many reoriented their projects to focus specifically on COVID-19. Throughout 2021, training and engagement continued virtually via Zoom, with six sessions delivered on various topics, including outbreak response, surveillance system evaluation and public health interventions. Trainees also presented their operational research projects to faculty and peers in an online forum.

The second face-to-face workshop included further training on outbreak investigation, data analysis and public health interventions. A COVID-19 intra-action review was conducted during this workshop, examining the contributions and roles of aFETPNG trainees in the national response (10). During the third workshop, trainees presented the findings from their intervention projects. FETPNG partnered with the Vital Strategies Data2Policy program to deliver a series of modules focused on translating evidence into public health policy. Using results from their field projects, trainees produced policy briefs for senior leadership in their provinces or at the national level. The final aFETPNG workshop also included an interactive expert-led workshop focused on sorcery accusation related violence, a significant public health concern in PNG (11). Throughout the program, each trainee was supported by a mentor during the face-to-face workshops and remotely during field placements. Mentorship was provided by national and international epidemiologists with expertise in disease surveillance, outbreak response, operational research and public health policy.

There have been relatively few published evaluations of FETPs globally (12). Existing evaluations have predominantly focused on outputs, such as the number of graduates, outbreaks investigated or surveillance systems evaluated. Some have included short-term outcomes, with relatively few have focused on medium- to longer-term outcomes and impacts (12). Responding to a growing interest in FETP impact evaluation, the Global Field Epidemiology Partnership (GFEP) identified impact evaluation as one of its four strategic priorities in March 2025 (13). Understanding how FETPs contribute to strengthening health systems and improving population health is critical for optimizing programs, advocating for program support, securing funding, promoting institutionalisation, and ensuring long-term sustainability.

This paper reports on a mixed-methods evaluation of aFETPNG. The evaluation examines the extent to which aFETPNG built epidemiology capacity, strengthened leadership within the national health system, and contributed to outbreak preparedness and response.

## 2 Methods

### 2.1 Evaluation setting

The evaluation was undertaken in Papua New Guinea (PNG), a lower-middle-income country in the Western Pacific region with a population of 11 million (14). PNG’s geographic diversity, including mountainous terrain, scattered islands and limited transport infrastructure, creates substantial challenges for delivering health services to remote communities. The country experiences a high burden of both communicable and non-communicable diseases and continues to face significant shortages in skilled health personnel, particularly in rural settings (15, 16).

### 2.2 Study design

For this evaluation, a convergent mixed-methods design (concurrent triangulation) was used (17). An essentialist or realist analytical approach underpinned both the quantitative and qualitative components, assuming that statistical findings reflect underlying real-world phenomena and that qualitative themes derived from explicit or semantic content represent a shared reality rather than socially constructed positions. Qualitative and quantitative data were collected within the same timeframe, analysed separately and then integrated (18, 19).

An impact evaluation framework (Table 1) developed specifically for evaluating FETPs was used to guide the evaluation (20). This framework is grounded in a general Theory of Change for FETPs and aligns with Kirkpatrick’s four levels of evaluation, assessing the learners’ response to a training program (Level 1: reaction – aligning to trainee outputs in Table 1), knowledge and skills gained (Level 2: learning - aligning with trainee and graduate outputs in Table 1), behavioural changes resulting from the learning (Level 3: behaviour – aligning with training and graduate outcomes in Table 1), and benefits at the organisational level (Level 4: results – aligning with public health system and community outputs, outcomes and impact in Table 1) (21). Level 4 was expanded to include the broader impacts on health systems and the community. Data were collected through a graduate survey, stories of change, and in-depth interviews with aFETPNG graduates, senior managers and health executives involved in the program.

**Table 1:** Field epidemiology training program impact evaluation framework.

Level of change	Outputs (products, projects, activities)	Outcomes (short and medium-term effects of program)	Impact (long-term effects of program)
Trainees	*Outputs during the training program* Trainees participate in competency-based Field Epidemiology Training Program and apply skills and knowledge.	*Short term outcomes achieved by trainees during training* Trainees are competent and committed to applying their skills and knowledge in their workplace.	
Graduates	*Individual outputs following graduation* Graduates develop and apply skills to strengthen disease surveillance, investigate outbreaks, conduct operational research and share findings through papers, reports and presentations.	*Short and medium-term outcomes by individual graduates* Skilled graduates strengthen public health activities in their workplace and contribute to a community of practice through alumni networks.	
Public health system	*Outputs affecting the wider public health system* Graduates embedded across all levels of the public health system, conducting projects and activities that strengthen public health systems. Graduates become junior FETP faculty.	*Short and medium-term outcomes affecting the wider public health system* Experienced Field Epidemiology workforce contributes to strengthening the public health system through routine application of knowledge and skills. FETP graduates support FETP as trainers and mentors.	*Longer-term effects of the program on the wider public health system* Strong public health systems across country. Strong and Sustainable FETP is established.
Community	*Outputs that affect the community* Community based public health activities and outreach programs conducted.	*Short and medium-term outcomes affecting the community* Improved access to higher quality public health services addressing priority community needs; community engaged in public health decision making.	*Longer-term effects of the program on the community* Improved public health realized through reduced morbidity and mortality from communicable and non-communicable diseases.

From Flint et al. (48).

### 2.3 Study objectives and key evaluation questions

The objectives of the study were to identify key outputs, outcomes and impacts of aFETPNG, assess the program’s effectiveness, and provide recommendations to improve the program. The evaluation addressed the following key questions:

1. What were the key outputs of the FETP?
2. To what extent did the FETP contribute to increased knowledge and skills of trainees and graduates?
3. To what extent did the FETP trainees and graduates apply their knowledge and skills into public health practice?
4. To what extent did the FETP contribute to a health system responsive to public and clinical health needs?
5. To what extent did the FETP graduates impact public health in the communities they serve?
6. To what extent did the FETP graduates contribute to the sustainability of Field Epidemiology Training Programs in PNG?

### 2.4 Sampling

All 17 aFETPNG graduates were included in the sampling frame for the graduate survey. For in-depth interviews, 13 graduates were purposively selected to ensure representation across geographic regions (Highlands, Southern, Islands, and Momase regions), levels of the health system (District, Provincial, National), and gender. All selected graduates were invited to participate. For interviews with senior managers/executives, participants were identified in consultation with the FETPNG Director and program faculty. All 8 senior managers/executives identified were invited to participate. Senior managers/executives did not complete a survey. The graduate survey and interviews focused on all 6 key evaluation questions, while senior managers/executive interviews focused on key evaluation questions 3–5.

### 2.5 Quantitative data collection

An online survey was distributed to all 17 aFETPNG graduates, with no exclusion criteria applied. The survey was administered in English via QuestionPro (2024 version) (22), and two reminder emails were sent to non-respondents at two-week intervals. Graduates provided informed consent prior to commencing the survey, which was completed from November to December 2023. Participants received a 50 Kina (~USD 12) data voucher to offset the high cost of accessing the internet in PNG. The graduate survey instrument is provided as Supplementary File 1. An additional online survey was administered to graduates of the intermediate FETPNG (iFETPNG) program, as part of a separate evaluation. As all aFETPNG trainees were graduates of iFETPNG, the iFETPNG survey enabled the collection of information from aFETPNG trainees who did not complete the program, providing insight into reasons for non-completion.

### 2.6 Qualitative data collection

Semi-structured interviews were conducted in person or via videoconference by trained interviewers from Australia, New Zealand and PNG. Interviews were conducted during September and October 2023. Written informed consent was obtained from all participants prior to commencement. All interviews were conducted in English, audio-recorded with consent, and supplemented with interviewer field notes capturing key reflections and stories of change. Interviews explored participants’ experiences of aFETPNG, including how graduates applied skills gained during and following the program. Graduates were also asked to share an example of one significant change they implemented or supported that could be attributed to their FETP training. Copies of the interview guides are provided as Supplementary File 2.

### 2.7 Data analysis

#### 2.7.1 Quantitative

Survey data were de-identified and analysed descriptively using Microsoft Excel (Version 16.98) (23).

#### 2.7.2 Qualitative

Interviews were recorded and transcribed verbatim by a professional transcription service. All identifying information was removed and transcripts were checked for accuracy by the primary author (JF). Transcripts were analysed thematically using a hybrid inductive-deductive approach described by Braun and Clarke (24) and Proudfoot (25). An essentialist, or realist, method guided the qualitative analysis, focusing analysis on the semantic content of interviews (24). Two researchers (JF and JW), an epidemiologist and a social scientist, immersed themselves in the data by reading transcripts multiple times and identifying initial units of meaning. Units of meaning were then grouped into categories to support data management and retrieval, and subsequently mapped to Kirkpatrick’s four levels (21). Both researchers independently coded a subset of transcripts and met to compare codes to ensure conceptual alignment. One researcher (JF) systematically coded the entire dataset supported by NVivo 14 version 14 (release 14.24.3) (26), giving full and equal attention to each transcript, with regular cross-checking meetings with the second researcher. Compelling quotations were identified for potential inclusion in the analysis report.

The primary analyst (JF) has extensive experience in field epidemiology workforce development and was directly involved in the design and delivery of aFETPNG. While this background provided valuable contextual understanding of the program, it also held the potential to influence interpretation of participant narratives. As part of a reflexive approach, JF documented his potential biases prior to commencing analysis and engaged in ongoing reflection throughout the analytic process. Coding decisions and emerging interpretations were discussed collaboratively with a second researcher (JW) who had no previous engagement with aFETPNG, or FETPs in general, to reduce the risk of interpretive bias.

### 2.8 Data integration

In line with the guidance of Fetters et al. (19), integration of quantitative and qualitative data was undertaken at the stage of interpretation and reporting. Survey findings and interview data were brought together to triangulate and verify results. Two complementary integration strategies were applied. First, *weaving* allowed qualitative insights to expand or clarify quantitative patterns. Second, *merging* enabled themes and survey results to be presented in parallel through side-by-side joint displays (27). Contribution analysis was used to assess the extent aFETPNG influenced the observed outcomes and impacts. Expected outcomes articulated in the theory of change were compared with changes reported through the qualitative and quantitative data (28). The integration of surveys, interviews, and stories of change enabled the demonstration of program contributions while acknowledging the role of wider contextual influences and other contributing factors (29).

## 3 Results

### 3.1 Quantitative

Thirteen of the 30 trainees (43%) commencing aFETPNG did not complete the program; 17 (57%) graduated. Of those who did not graduate, nine provided reasons for not continuing with aFETPNG. The most common reason (67%, *n* = 6) was the increased workload due to the COVID-19 pandemic. Other reasons included personal factors (33%, *n* = 3), being busy with work other than COVID-19 (22%, *n* = 2), not getting along with their aFETPNG mentor (11%, *n* = 1), and starting another training program (11%, *n* = 1).

All (100%) of the 17 aFETPNG graduates completed the online survey. There were 10 male and 7 female respondents. Most graduates (*n* = 15, 88%) were mid-career health professionals aged 30 to 49 years, and the majority (*n* = 15, 88%) were employed as Health Extension Officers at the time of completing the survey. In PNG, Health Extension Officers are clinically trained health managers, often responsible for managing health centres and/or health programs. All graduates had more than five years of experience working in a health-related field prior to enrolling in aFETPNG, with almost half (48%, *n* = 8) having more than 15 years’ experience.

When asked about their highest level of education, 29% (*n* = 5) of graduates indicated holding a university or college certificate or diploma, 35% (*n* = 6) a university degree, 24% (*n* = 4) a postgraduate diploma or certificate, 6% (*n* = 1) a Master’s degree, or 6% (*n* = 1) a medical degree (MBBS). Most aFETPNG graduates (*n* = 10, 59%) were working at the provincial level, with 24% (*n* = 4) working at the district (sub-provincial) level, 12% (*n* = 2) at the local (sub-district) level, and 6% (*n* = 1) at the national level. At the time of survey, almost all graduates (*n* = 16, 94%) were employed by the government sector, with one (6%) working for PNG’s Church Health Services. Of the graduates surveyed, 12% (*n* = 2) were senior or executive-level managers, 41% (*n* = 7) were program managers, and the remainder (47%, *n* = 8) worked in clinical or technical roles.

### 3.2 Qualitative

Semi-structured interviews were conducted with nine of the 13 (69%) aFETPNG graduates identified for potential interview and all eight (100%) senior managers and executives. Of the graduates interviewed, four were male (44%) and five were female (56%). Graduates represented all four regions of PNG (Highlands, Southern, Islands, and Momase) and worked at the national (*n* = 3, 33%), provincial (*n* = 2, 22%), and district (*n* = 4, 44%) levels. The senior managers and executives represented the national (*n* = 3, 38%) and provincial (*n* = 5, 63%) levels. All senior managers and executives interviewed were male.

Three themes emerged, aligning with the 4 levels of the Kirkpatrick evaluation method; (i) reaction (Kirkpatrick Level 1), (ii) learning and behaviour (Kirkpatrick Levels 2 and 3), and (iii) results (Kirkpatrick Level 4).

### 3.3 Kirkpatrick level 1: reaction

Graduates reported predominantly positive reactions to the learning experience (Kirkpatrick’s first level of evaluation) (Table 2). The majority (≥88%) rated the training content, materials, and delivery methods highly, reflecting strong engagement with the core components of the program. In contrast, mentoring support between workshops received the lowest ratings, indicating an area where participants perceived less value or consistency.

**Table 2:** Graduate reactions to different components of learning during aFETPNG training, Papua New Guinea, 2023 (*n* = 17).

Training component	Good	Average	Poor
The training content	17 (100%)	0 (0%)	0 (0%)
The training materials provided	15 (88%)	2 (12%)	0 (0%)
How the training was delivered	15 (88%)	2 (12%)	0 (0%)
The knowledge of the facilitators	14 (82%)	3 (18%)	0 (0%)
Mentoring during workshops	13 (76%)	4 (24%)	0 (0%)
Mentoring between workshops	12 (71%)	5 (29%)	0 (0%)

During interviews, graduates recommended having more time scheduled for mentoring during workshops. The importance of having one assigned primary mentor was also highlighted, with several participants noting that receiving advice from multiple mentors sometimes generated conflicting messages and uncertainty about the best course of action. Graduates also recommended pairing junior mentors with more experienced mentors to support the development of their technical competence and confidence. The work-integrated learning model and the structured field projects were identified as crucial components for consolidating skills and providing hands-on experience. As one graduate reflected, “the field assignments were the turning point for me; I learned more solving real public health problems than I ever did through lectures alone” (G1).

### 3.4 Kirkpatrick levels 2 and 3: learning and behaviour

#### 3.4.1 Skill development and application

Graduates reported a high level of confidence and frequent application of key field epidemiology skills. The majority indicated being very confident in identifying unusual events from surveillance data (82% very confident; 94% applied), using surveillance data to guide programming (88% very confident; 94% applied), conducting outbreak investigations (88% very confident; 82% applied), and implementing evidence-based public health interventions (76% very confident; 76% applied) (Table 3). These findings suggest that the program was effective in developing foundational field epidemiology competencies. Graduates expressed lower confidence and less frequent application of more complex or specialised skills, including evaluating surveillance systems (59% very confident; 47% applied), designing and conducting operational research or epidemiological studies (29% very confident; 35% applied), delivering oral scientific presentations (41% very confident, 47% applied), and evaluating public health interventions (47% very confident; 53% applied). Activities linked to research, policy, and preparing abstracts or peer-review papers were associated with the lowest levels of confidence and application.

**Table 3:** Confidence in, and application of, key field epidemiology activities among aFETPNG graduates from Papua New Guinea, 2023 (source: graduate survey).

Field epidemiology-related activity	Confidence (*n* = 17)			Application (*n* = 17)		
	Very confident	Confident	Not confident	Yes	No	Do not know
Creating and managing a line list	16 (94%)	1 (6%)	0 (0%)	15 (88%)	2 (12%)	0 (0%)
Using surveillance data to guide public health programming	15 (88%)	2 (12%)	0 (0%)	16 (94%)	1 (6%)	0 (0%)
Conducting an outbreak investigation	15 (88%)	2 (12%)	0 (0%)	14 (82%)	3 (18%)	0 (0%)
Summarising an outbreak investigation in a written report	15 (88%)	2 (12%)	0 (0%)	14 (82%)	3 (18%)	0 (0%)
Identifying unusual events from surveillance data	14 (82%)	3 (18%)	0 (0%)	16 (94%)	1 (6%)	0 (0%)
Implementing an evidence-based public health intervention	13 (76%)	4 (24%)	0 (0%)	13 (76%)	4 (24%)	0 (0%)
Conducting descriptive data analysis and interpreting results	12 (71%)	5 (29%)	0 (0%)	14 (82%)	3 (18%)	0 (0%)
Evaluating a surveillance system and making recommendations	10 (59%)	6 (35%)	1 (6%)	8 (47%)	9 (53%)	0 (0%)
Evaluating a public health intervention	8 (47%)	9 (53%)	0 (0%)	9 (53%)	8 (47%)	0 (0%)
Giving an oral scientific presentation	7 (41%)	8 (47%)	2 (12%)	8 (47%)	9 (53%)	0 (0%)
Designing and conducting operational research/epidemiological studies	5 (29%)	12 (71%)	0 (0%)	6 (35%)	11 (65%)	0 (0%)
Developing evidence-based policy recommendations	2 (12%)	13 (76%)	2 (12%)	5 (29%)	12 (71%)	0 (0%)
Writing an abstract for a scientific conference	2 (12%)	13 (76%)	2 (12%)	6 (35%)	11 (65%)	0 (0%)
Writing a scientific manuscript for a peer-reviewed journal	1 (6%)	9 (53%)	7 (41%)	2 (12%)	15 (88%)	0 (0%)
Creating a policy brief	1 (6%)	13 (76%)	3 (18%)	5 (29%)	12 (71%)	0 (0%)

When asked whether covering some topics more thoroughly, or including additional topics, would have strengthened their learning, 82% (*n* = 14) of graduates said yes. The areas most frequently identified for further development included data analysis software (*n* = 7, 50%), scientific writing (*n* = 4, 24%), and policy development (*n* = 4, 24%).

Almost all graduates (*n* = 16, 94%) were employed in roles requiring field epidemiology skills, and all (*n* = 17, 100%) reported using data to inform their decision-making and to support decision-making by others. Almost all (*n* = 16, 94%) indicated that their work directly contributed to the development and implementation of public health projects and programs, and 76% (*n* = 13) reported influencing the development of health policies. Beyond applying field epidemiology skills, all aFETPNG graduates (*n* = 17, 100%) reported transferring knowledge to colleagues through mentoring or other forms of professional support. Graduates also actively disseminated findings from their field epidemiology-related activities, including through presentations to colleagues and supervisors (*n* = 16, 94%), to community leaders and members (*n* = 6, 35%), at national meetings and conferences (*n* = 6, 35%), and/or at international forums (*n* = 3, 18%).

When asked about aFETPNG skills that were rarely or never used, graduates most frequently identified operational research and the development of policy briefs. In addition to feeling less confident in these competencies, limited time, competing work demands, and resource constraints acted as barriers to applying these skills in practice.

Graduates highlighted the importance of interpersonal skills and leadership capabilities developed through aFETPNG. They reported improvements in public speaking, community engagement, training and facilitation, program management, and leadership (Table 4). These changes were often noted by colleagues and managers. One graduate commented: “My colleagues saw the positive impact the training has had, and they all want to come and do FETP… My colleagues and everybody are asking, ‘How did you learn that? Where did you learn that?’… They are beginning to see that this is something that can have impact” (G1).

**Table 4:** Development and application of interpersonal skills from aFETPNG training among graduates, with key themes and quotes from graduate interviews, Papua New Guinea, 2023.

Skill	Supporting quotations
Public speaking	“I presented about TB surveillance in my province at an international TEPHINET conference in Cambodia. When I came back, I presented that same research paper at the province level and then I was asked to conduct a province-wide evaluation for the TB program.” (G2) “I presented my research outcomes to all the senior management, and they were quite impressed.” (G3)
Community engagement	“Convincing rural communities about preventive measures was challenging. Using the communication techniques I learned in FETP, I could tailor messages to their cultural context.” (G4) “Due to the misinformation on the COVID-19, we involved social mobilizers. And we talked to the communities… so that people can be able to access the services that we are bringing to them. And sometimes we face challenges. People are not on our side, they do not want us in the community, but we have to penetrate these communities by providing education, communicating with them, telling them the benefits, and [addressing] some of the misinformation going around also. So involving trusted people, like community leaders, some people are willing to work with us and listen to us; we work along with those people, not the ones who are trying to go against us.” (G6)
Training	“I’m proud to [be] providing these specific skills to equip and enhance our existing health workforce to strengthen and improve surveillance activities… to respond more effectively to outbreaks and any other public health emergencies.” (G2) “I run internal refresher training for staff, especially to keep an eye and watch over if there are any outbreak cases coming in.” (G5)
Management	“There’s been a lot of training conducted on a lot of management skills…and they all have shaped me into who I am today. So I think the trainings that I received, gave me a promotion to be where I am. And also human resources. The conflict and resolution skills as well. Dealing with human issues is quite complicated. So I’ve learned a lot from this area as well. It has enabled me to respond.” (G3) “With time management, I would say FETP really, really shaped me and moulded me professionally. And personally I would say, it really enhanced my work.” (G2)
Leadership	“This FETP program has helped me in terms of confidence, communication, but I also think that FETP training itself mirrors the way we show leadership… FETPs not only help us to analyse data… but also to become good leaders in their place of work.” (G6) “One of the things that I learned from field epi is leadership skills. Understanding our colleagues, how to work with them, identifying their strengths and weaknesses, and helping them in the areas that they have strengths, and assisting them in the areas that they are weak, and to show them where to go.” (G4)

#### 3.4.2 Enablers and barriers to applying FETP skills

Interviews identified several enablers and barriers which supported or hindered graduates in applying their field epidemiology skills in their respective workplaces. These were organised into the following themes: professional networks, workplace, gender, security, and professional jealousy.

The role of supportive *professional networks* in enabling the application of FETP skills was strongly emphasised. Graduates described the FETP alumni network as a central source of trusted expertise and problem-solving support: “The network of FETP alumni has become my go-to resource. Whether I need advice on data analysis or outbreak control measures, I can count on a colleague to help” (G7). Engagement with local health workers and community leaders also created a conducive environment within which to apply their skills. One graduate highlighted how community engagement provided access to and support from political leaders: “When you have a good communication with community leaders at the community level… they communicate with the parliamentary member and tell him, this is the kind of person he is, and he wants to do this and that. So then the member himself communicates with them directly” (G8).

Supportive managers and colleagues in the *workplace* encouraged the use of field epidemiology skills and built the confidence of graduates. As one graduate stated, a supportive workplace “helped motivate and boost morale” (G2). In some cases, recognition of new skills generated numerous opportunities to apply them: “Since I graduated, everybody just throws things at me… I just have to do it, the province is depending on me” (G3). Conversely, workplace cultures that failed to acknowledge or value the skills of graduates undermined their confidence and served as a barrier to the application of their skills. When managers did not act on data or recommendations generated by graduates, they felt demoralised. When this happened, one graduate said: “I feel that this training has not been valued, whatever I have to offer in this place of work has not been valued. So it’s demoralizing.” (G5). Heavy workloads and tight schedules were additional barriers, making it difficult to find focused time to apply new skills: “Many things come in, they bombard me every day. So it’s all about time, that’s the barrier. I can only do so much” (G4). Limited or delayed access to financial, logistical, or human resources also restricted the application of field epidemiology skills and the implementation of projects. Graduates described delayed release of funds, restrictions on fuel for fieldwork, limited phone credit for reporting, and a lack of trained staff to support outbreak response as key constraints.

Experiences relating to *gender* varied. Some graduates reported gender-based barriers to leadership roles: “Even though you have that knowledge and skills, but because you are female, you may not be given the position … some managers, they still have this mentality” (G2). Others did not experience barriers related to gender: “Personally, I didn’t see any impact of being female, at the workplace and also at training” (G5).

Graduates reported difficulty travelling to collect data or conduct outbreak investigations due to *security* concerns arising from tribal conflict or criminal activity. At times these security risks prevented graduates from implementing or completing field epidemiology projects, serving as a barrier to the application of their skills. One graduate stated that youth gangs, “might like to come and stop the vehicle, it has become a norm… So it’s not really easy to control them… even my offices have been attacked” (G8).

Several graduates experienced *professional jealousy* from peers and superiors, particularly when their new expertise was perceived to challenge existing hierarchies. This was reported as a barrier to applying skills: “I am just an HEO (Health Extension Officer), and they are doctors above me. They can’t accept someone like me to run the show and get things right” (G3). Another graduate recalled: “They do have this jealousy, professional jealousy. When I went back to that district and applied the skills, even my District Health Manager, she was threatened with her position because of my performance in that district… When you are producing results, this may cause ill feelings among your colleagues or your managers” (G2).

Graduates proposed several strategies to strengthen support for applying FETP skills following their training. Continued advocacy to provincial health authorities (PHAs) was recommended to ensure organisational support and recognition during and after training: “Not all PHAs are supporting their fellows, we need to continue to advocate through executive management… there are a lot of good stories and impact… we need to [share this] as part of our advocacy to the executive management” (G2). Graduates also emphasised the importance of continuous learning, refresher courses and ongoing mentorship to support their daily practice: “The faculty should continue mentoring the FETP graduates to do more research” (G8). Graduates suggested creating a formal FETP Alumni Association to support the ongoing application of skills. Others appreciated the value of existing e-learning modules for supporting their learning following graduation: “The e-learning modules, I see that it is very important, [I recommend] we complete them and then send them back to you for assessment” (G8).

### 3.5 Kirkpatrick level 4: results (outputs, outcomes, impact)

All graduates emphasised the role of aFETPNG in enhancing their capacity to conduct epidemiological tasks, including disease surveillance, outbreak and emergency response, data collection and analysis, and health programs delivery. The outputs, outcomes and impacts described by graduates and senior managers are discussed below and summarised in Table 5.

**Table 5:** Integration of quantitative and qualitative findings to demonstrate outputs, outcomes and impact from applying skills among aFETPNG graduates, Papua New Guinea, 2023.

Skills	Quantitative results	Supporting quotations from graduates	Supporting quotations from senior managers and executives
Disease surveillance skills	From the 17 survey respondents: 59% (*n* = 10) evaluated a surveillance system (or component of a surveillance system) 47% (*n* = 8) made an improvement to a surveillance system 41% (*n* = 7) used surveillance data to detect an outbreak 82% (*n* = 14) analysed data from a surveillance system 82% (*n* = 14) used surveillance data to guide the delivery of a public health program or activity	“I knew nothing about surveillance; when I attended this FETP I learnt about disease surveillance and responding to outbreaks. I learned a lot, in fact, I am doing it right now; indicator-based surveillance, event-based surveillance and syndromic surveillance… From this surveillance, it is helping us identify outbreaks. We have already established a surveillance system in the district… I do the investigation and do the reports and provide to management for action… I am confident with my work… We have strengthened the surveillance system… I contribute a lot to decision making… We now have a surveillance network with officers on the ground. I think we are doing fine after the training.” (G9) “I was doing little surveillance, but after that first workshop on surveillance, I really did active surveillance. I conducted district surveillance training. Even the WHO consultant that was assigned to be at the province, he came down for that training; he said, “you are way ahead of the other districts, other districts are not doing it, and you are doing it”…. After FETP, I started a training series for frontline nurses. We covered everything from data reporting to community outreach, which had an immediate impact on surveillance quality.” (G2) “When we had COVID pandemic, I was given a position for surveillance lead… I was the lead and I managed data analysis, reporting, and also the outbreak investigations. I was able to lead a team and apply the steps that we were taught in FETP… We do monthly reports, and weekly sitreps for COVID… and based on that, the decisions were made by the National Contel Centre… based on these reports, they had to call our emergency lockdown… So the data we were reporting did have an impact on the decisions… We did house to house contact [tracing], and we did home surveillance… And then the numbers started to decline after we did some recommendations.” (G1)	“You know in the past [Provincial Health Authorities] did not really see the importance of the field epidemiologists. But now… we have seen the importance of excellent surveillance and disaster relief. And we are now trying our very best to put one [field epidemiologist] in every district…there is a big need now for these surveillance officers or field epidemiologists.” (SME1) “The graduates are actually doing a good job. One is doing daily surveillance. One is responding to events under surveillance. They’re reporting to the WhatsApp group that they formed, and that’s how we monitor what’s going on… [they] are currently very active in doing surveillance after training.” (SME2) “I feel that routine surveillance system and public health event detection is crucial, because as you know, when there’s an event… and an investigation, the intervention is done by FETP graduates… [they] improve the routine reporting, surveillance and verification of events… using of data to make decisions… The first ever field epidemiologist position [was] created in the PHA in Eastern Highlands… that is one of the best provinces now in terms of realizing the importance of surveillance and also the field epidemiologist playing a role in leading that part of the surveillance… FETP is driving the recognition that we need to have surveillance officers in the system to assist, so we can overall improve the surveillance system.” (SME3)
Outbreak investigation and emergency response skills	From the 17 survey respondents: 82% (*n* = 14) of graduates investigated one or more outbreaks as lead or support investigators 18% (*n* = 3) had not investigated an outbreak since completing aFETPNG. Graduates investigated 55 outbreaks ○ 37 as lead investigators ○ 18 as support investigators Graduates investigated an average of 1.8 outbreaks per year 18% (*n* = 3) of graduates were deployed outside their usual province of work to support an outbreak response 71% (*n* = 12) led the writing of an outbreak report 82% (*n* = 14) contributed to the writing of an outbreak report 100% (*n* = 17) of graduates led community engagement activities during the COVID-19 88% (*n* = 14) led community engagement activities during non-COVID outbreak investigations.	“With FETP and the skills gained in outbreak investigation…it really taught me and I can lead that district, and I did lead during that pertussis outbreak.” (G2) “We developed skills to participate in outbreak investigations from the FETP training. In most of the outbreaks… I am always the team leader because of the FETP skills… Recently this year I participated in one of the health events that posed a threat to the people along the river…because of some chemical. People were afraid to eat the fish in the river, they saw that the fish were just dying from the river. And then they saw the sores on the fish were actually like what appeared on the human body. So people were in fear. This was one of the outbreak investigations I participated in after the FETP program.” (G8) “I was engaged to conduct emergency research during the pandemic period because the testing rates were very low. Even though healthcare workers were trained to do COVID testing, not many tests were done. So we wanted to find out why? I coordinated the research, supervising the interviews, doing phone calls. And then I was taking lead in doing the analysis and also report writing. One of the successes was that we published a paper… we published in the International Journal of Infectious Diseases.” (G2) “I actually led the investigation of the first COVID-19 case in [province], case number 8 for the country, and I was able to contain this first case, and later, manage a cluster, and then community transmission. It was then management realized the importance of surveillance and field epidemiology. Management now is fully supporting surveillance activities and rapid response. We were given an office, we were given office equipment, we now have a surveillance network with officers on the ground. I think we are doing fine, after the training especially… Before, outbreak investigations were not done. [FETPNG] has really changed my work… The major aspect I was involved was COVID-19, everyone was impressed, even the clinicians. I took the lead, I did the case management and outbreaks, we gained respect after COVID-19… What we are doing from the FETP is much better, this is why people respect us.” (G9)	“You know, a lot of our workforce wanted to just leave [during COVID-19], they just wanted to walk off from work. So [the FETP graduate] was appointed to take charge… he was doing all the things, even clinically in the ward, and also the surveillance. He was also overseeing everything else.” (SME4) “I sent [graduate] to investigate water related diseases and some of the issues that were popping up as an after effect of tribal fighting, burning down of houses…the people were displaced… all these things he responded to. So I realised the importance of these guys, they are very important in our organisation and in the field…Also, using the [graduates’] report, we asked partners to support us to renovate and provide a respiratory centre for screening, testing, and vaccination of COVID-19, not only for COVID-19, but any respiratory disease, and then getting the specimen, sending to a public health lab.” (SME5) “Public health events, including outbreaks, are now being led by [graduates]… if it is something they can handle, they handle it, and then they share the information with us. For example, in Bougainville for the volcano eruption, our FETP graduate is the team leader in that disaster management and disaster coordination and doing surveillance work with the displaced population… we are trying to… make them leaders in the Rapid Response Team.” (SME3)
Data collection and analysis skills	From the 17 survey respondents: 88% (*n* = 15) improved data management in their workplace 94% (*n* = 16) improved data collection in their workplace 82% (*n* = 14) improved data analysis and interpretation in their workplace 82% (*n* = 14) improved report writing in their workplace	“Previously, we relied on assumptions. After FETP, I learned to use data to guide decisions. The FETP program has given us a lot of confidence, a lot of skills, especially to collect data, analyse data, interpret data, and report findings, then trying to understand potential outbreaks and attending to the outbreak… we can help [management] a lot, so they make their decisions based on the evidence base…There is demand for data analysis, management are asking me to do analysis for vaccination and other programs… when they go to meetings in Moresby, they use our information to present our data. They value it very much…they really understand what will happen when they support us in our program.” (G9) “I never thought I would be comfortable using statistical tools, but now I’m able to run analyses and confidently present findings to policymakers… FETP has equipped me to realise and appreciate the importance and value of data… when we see that raw data, it does not really give us a meaning. But when we are putting them into graphs and tables, it really speaks for itself and really gives us the picture… there is a pattern, there is a story, there is an information behind the numbers… I really applied those skills after the training, it really opened up my mind… we have got a lot of data sitting here, we need to analyse those… the solution is here, we just need to utilise these data.” (G5)	“He learned a lot from the training in terms of dealing with the data and the analysis of the data… Now he is able to use the data and analyse it and get the outcome from what the data is telling him.” (SME6) “We at the executive level, and at the management level, we do not want to act without evidence, we may be putting money in the wrong place.” (SME7) “The most valuable skills from the program is the ability to collect, analyse, and interpret data and to do presentations and develop reports and plans… [Graduate] has also been utilising his skills and knowledge, using data to put in place operational plans… And he was invited by the member for [region], who is also the communication minister, to come to the national level and present his reports at a national level.” (SME8)
Health program and health system strengthening skills	From the 17 survey respondents: 65% (*n* = 11) supported a research project (as part of the research team but not the lead) 35% (*n* = 6) led an operational research project (as the lead researcher) 76% (*n* = 13) implemented an evidence-based intervention	“FETP taught me the skills and knowledge I have applied. It also helped me to look at the program data… to see where to develop some interventions to address what I can do at my level… [so] I can contribute to make some difference.” (G2) “From the skills that I learned from FETP, I managed to open some health posts at the primary level with assistance from the district development authority, [I used] partnerships skills to influence them, and those skills [were gained] through the FETP training… I sat down, held some short meetings with them, and asked them for funding. So they helped me. We went down to the community level and opened several aid posts which are now operating and providing services to the people… the partnership skills, those I learned from FETP.” (G8) “I did my operational research on vaccine preventable diseases… I found out that most healthcare workers are not well invested in immunisation and the caregivers do not understand why they are bringing the children for vaccinations. So I created teams to go out and do awareness. I presented [my findings] to the technical working EPI group [and] our partners…to fund everything and work together. We train staff… we do it twice each year… So far so good. It’s been continuing for the last 3–4 years… there’s quite a lot of improvement.” (G3) “In previous years, our [district] was ranking always at the very bottom level… When I was given that opportunity to manage the district, health partners could literally see the change in the certain programs. One of the significant things that I am really proud of today, is the achievement in the recent measles, rubella and polio campaign… we ranked number four in the country out of the 89 districts, and we were first in Momase region and we were first in the province. So this was one of the significant things which I’m very proud of, because of the FETP training that gave me these skills and knowledge to use them in the field. Most of my six health facilities, they have even performed more than 95% of the target that has been set by the National Department of Health.” (G8)	One of the [advanced graduates] has done operational research… So the FETP skills and knowledge continues to be alive… I have a lot of confidence that… we will be able to utilise these graduates to become trainers so that we can separately conduct FETP training back in a location and place where we are.” (SME8) They actually put a lot of influence in their place of work, there’s a lot of change. What they are very, very conscious about is data…. collecting baseline data, making the analysis, seeing the performance level, and then based on that, they put in place programs. So that helps to uphold and improve indicators of programs.” (SME4) I’ve been practicing in some remotest part of the country. I’ve managed epidemics, malaria outbreaks, flu outbreaks, diarrhoea outbreaks, [we need to] pick up numbers very early, and then start doing prevention, because once it goes into the population, it’s like a bushfire and to extinguish it, it’s going to be difficult. So we have got to pick these patterns quickly… we at the executive level in the management level, we need those kinds of evidence. Without evidence, we may be putting money in the wrong place. We may be making poor decisions.” (SME7)

#### 3.5.1 Disease surveillance

Most graduates reported being involved in analysing surveillance data (*n* = 14, 82%) and using surveillance data to guide decision-making (*n* = 14, 82%), including for the detection of communicable disease outbreaks (Table 5). Interview findings highlighted how aFETPNG consolidated and extended the surveillance capabilities gained during the intermediate FETPNG program. Graduates described becoming more proactive in promoting and supporting surveillance following aFETPNG, including training data providers. Several noted having leadership roles in surveillance during the COVID-19 pandemic.

Graduates provided numerous examples of longer-term surveillance outcomes resulting from their work. For example, a hospital-based surveillance initiative led by a graduate resulted in the addition of new domestic violence indicators into the national surveillance system. Surveillance tools developed during COVID-19 continued to be used following the response. One graduate developed, “a mobile-friendly app where instead of using the paper, the case investigation form is formulated on an app” (G1). Multiple other provinces contacted this graduate wanting to use this app: “They wanted that app to be used in their provinces, because the outcome was that it improved reporting, and the timeliness of reporting… There was a lot of interest from other provinces, [and] not only for COVID, but for other diseases too” (G1). Another graduate described initiating the development of a software app to capture real-time surveillance data from remote areas using satellites and smartphones. The graduate linked this initiative to their aFETPNG training: “One of the things that FETP has given me is the knowledge and skills, and now I am negotiating with one of the software engineers” (G8).

#### 3.5.2 Outbreak investigation and emergency response

Graduates surveyed collectively investigated 55 outbreaks following aFETPNG (Table 5). The majority (*n* = 14, 82%) reported involvement in one or more outbreak investigations as a lead or support investigator, while three (18%) had not participated in any outbreak investigations following their training. The different types of outbreaks investigated are shown in Supplementary Annex B. Interview data demonstrated that aFETPNG was perceived as pivotal in equipping graduates with the confidence and technical capability to undertake outbreak investigations. As one participant explained, “the training provided a lot of strength and knowledge and skills to do surveillance and investigations” (G9). Graduates described systematically applying outbreak investigation approaches across diverse contexts: “I can quickly identify cases, define the outbreak, and mobilize a team for immediate action” (G3), and “I have been involved in contact tracing, sample collection, case management, community risk communication, case detection, active case finding” (G5). Many assumed leadership roles during the COVID-19 response.

Graduates also described a range of disease control measures they implemented to control disease transmission, including public awareness on the radio, immunisation campaigns, and the installation of rainwater tanks to provide potable water. One graduate highlighted a community-led approach to implementing control measures. Using skills from their training, they engaged with affected communities when traditional control measures failed: “We have mosquito net distribution… we have clinical treatment…but why do people still get malaria? I decided it’s best that I go and sit down with the community and let the community tell me how they think they should prevent themselves from getting malaria” (G8).

#### 3.5.3 Data collection and analysis

Most graduates surveyed reported being able to apply their aFETPNG-acquired skills to improve data collection (94%, *n* = 16), data management (88%, *n* = 15), and data analysis (82%, *n* = 14) (Table 5). Interview data reinforced the central role of aFETPNG in strengthening analytical capability, with one graduate explaining that the most valuable skills gained through aFETPNG were those related to, “data management, collecting data reports, analysing data, interpreting the data and trying to draw insights into what the data means and what actions we should take” (G6). Graduates reported that increased proficiency in data analysis enhanced credibility and trust within their organisations, improving their influence in decision-making processes.

A number of graduates described how they used their data skills to build the capacity of others. One graduate observed that many frontline officers responsible for key public health programs such as TB, HIV, and maternal and child health, lacked skills in analysing program data: “After going through the training, I realised that many of the officers in facilities do not know how to analyse data” (G4). After raising this concern with senior management, the importance of these skills was acknowledged by the Director of Public Health as, “something that every Officer in Charge of the facility must know”. The graduate subsequently trained health workers to routinely record and analyse data to guide the delivery of their programs: “The information must be analysed so it tells them where they’re going… every data must be recorded and placed on the board in a graph form for them to see”. Recognising the value of this initiative, the graduate’s manager authorised funds to scale up the training across the district, stating that, “once you’ve finished with the district, then you go district by district in the province” (G4).

#### 3.5.4 Health program delivery

Sixty-five percent (*n* = 11) of aFETPNG graduates reported involvement in operational research aimed at strengthening health programs or health systems (Table 5). Many of these initiatives were continuations or expansions of field projects initiated during aFETPNG. Graduates described a range of projects and public health interventions related to disease prevention and control programs (e.g., TB, HIV, COVID-19), non-communicable diseases, water and sanitation, and routine immunisation. Interview findings highlight how aFETPNG equipped graduates with skills to strengthen these public health programs using evidence-based strategies. Graduates spoke of engaging with community stakeholders and securing financial and human resources to strengthen public health programs.

Graduates provided multiple examples of programmatic improvements resulting from their work. These included increased TB and HIV notification rates and improved treatment outcomes through, “extending outreach programs into the community, doing mobile clinics, and combining TB and HIV, together” (G2). One graduate reported improving TB treatment success rates from 80 to 93%, describing this as, “a very significant achievement for the province… we achieved 93% treatment success rate for all TB cases in 2022, and then each quarter for this year 2023, we are also seeing that has been maintained over time” (G6). This improvement was directly linked to training: “That’s because of FETP training, giving me the skills to know how to deal with program data” (G6). Other graduates described strengthening TB and HIV services by establishing a TB and HIV screening clinic at the district level rather than requiring patients to travel long distances to a neighbouring health centre. Field epidemiology skills were also used to improve infection control practices in health centres, including during COVID-19. One graduate described pregnant women and mothers with infants coming for vaccination and waiting alongside suspected COVID-19 cases in the hospital outpatient department. They stated that, “the skills and knowledge that I acquired through the FETP training [were] helpful to improve processes [and] the services in the facilities” (G5), resulting in infectious and non-infectious patents being physically separated while waiting in the outpatient department. Graduates also used operational data to inform service planning and resource allocation. One example involved reviewing caseloads across health facilities and collaborating with partners to open new aid posts to expand access to care. After establishing several new posts, the graduate noted a reduction in caseload pressures at district-level facilities.

#### 3.5.5 Professional outcomes

Graduates reported a range of broader (non-technical) professional outcomes resulting from their participation in aFETPNG. Almost all (*n* = 16, 94%) indicated that the program increased their confidence in the workplace. Interview data illustrated the depth of these outcomes. One graduate explained: “I can say that the FETP program has given us a lot of confidence” (G9). Others described significant personal and professional growth: “I was a nobody when I was a clinical HEO, but after being in the program, I can now do presentations, stand confidently and speak to the crowd” (G4); “I would say [FETPNG] brought me out from my place of being quiet… It has built the confidence to make decisions and even make recommendations where management are now actually recognising it” (G1).

Graduates also reported strong outcomes in leadership, communication, and management capability: “Leadership skills are one of the very important components that I’ve learned in field epi, which has helped me to be where I am now and is still pushing me up” (G8). Enhanced communication skills strengthened workplace relationships and influence: “The training had a very great impact on me personally and in the work I do with my team… I learned the communication strategies… I see that officers begin to really appreciate the way I respond to them” (G8). Others highlighted the respect they gained from colleagues: “When I talk, people respect me, I think this is a big change I’ve seen since FETP” (G3). Another described wide-ranging professional impacts: “Personally has changed me a lot, in terms of management, to manage my time, to become a team leader. It has helped a lot of things in my life, my family, my workplace, my colleagues are starting to respect me as a field epidemiologist, some of the work I am doing they are really appreciating. It has changed my life” (G9).

Almost half of the graduates (*n* = 8, 47%) received a promotion after completing aFETPNG and all 100% (*n* = 8) reported that aFETPNG was very important in achieving this advancement. Just over one-third (35%, *n* = 6) commenced or completed further study following aFETPNG, including four Graduate Certificates (67%) and one non-accredited training activity (33%). Interview findings reinforced the critical role of aFETPNG in creating pathways for career progression. One graduate described recognition by senior health officials based on performance improvements following training: “Based on my performance in the district, he wanted to give me a position at the provincial level… the CEO looks on my district as a role model, [he] always uses us as an example to other districts; it is only the FETP skills that I have gained that enabled me” (G8). Similarly, a graduate in a different province was asked to, “climb up higher to be provincial disease controller manager” (G4) because of their competence in data analysis and reporting. This graduate reflected: “I’ve realised it is a very impactful and very good training that I received that has impacted my life so much” (G4). Another graduate described, “being part of [a WHO] training team to support Solomon Islands… that’s a big contribution because of the FETP training” (G6).

#### 3.5.6 Contribution to working groups and FETPNG

Almost one quarter of graduates (*n* = 4, 24%) reported contributing to a working group or committee at the national, regional, or international level since completing their FETP training. These included a national research advisory group, a regional FETP manual working group, and the national FETPNG faculty. Nearly half of advanced-level graduates (47%, *n* = 8) went on to serve as FETPNG trainers and mentors. Of these, five (29%) contributed to the Frontline FETP at the provincial level, and four (24%) to the Intermediate FETP at the national level.

#### 3.5.7 Observations and recommendations from senior managers and executives

Senior managers and executives interviewed spoke highly of aFETPNG, the skills of graduates and the contribution of graduates to public health practice. They emphasised the rigorous nature of the program, noting that graduates, “have been through a lot of mentoring; they have been through a lot of coaching and teaching” (SME8). Improvements in performance at the district level were frequently attributed to the training: “Since we started in 2023, I can see the performance has improved at the district level; it reflects the FETP training” (SME4). Senior leaders valued a wide range of field epidemiology competencies, including, “the ability to collect, analyse, and interpret data, do presentations and develop reports, plans and budgets, and also… confidently carry out outbreak investigation” (SME8). Others stated: “FETP training is adding value… It’s equipping [graduates] with knowledge on epidemiology; that’s the part that has been missing in the medical schools” (SME5), and “You don’t get this in the university or college; this kind of skill-based study has not been taught anywhere in any college that we have in PNG” (SME3). The results produced by graduates, particularly during COVID-19, had strengthened organisational support for the program: “Senior management are aware that the training is useful, they’ve seen the result of it in the COVID response… we have the support [for FETPNG] in the management department, we have the support in the Public Health Authorities” (SME3).

Senior managers and executives described substantial contributions of aFETPNG graduates to disease surveillance, outbreak response, and health system strengthening. Surveillance was identified as a critical priority: “I see [surveillance] as the number one priority for FETP, because I can’t see any other way to improve surveillance” (SME3). Improvements were also observed across investigation, reporting, and control activities: “I could see there’s an improvement in the area of investigation, prevention and control, and also there’s improvement in how they structure their reports… I could see a big difference” (SME4). In one example, a manager spoke of the graduate’s role in a very large methanol poisoning incident which resulted in significant morbidity and mortality. The graduate, “took control of the public health education awareness, collecting samples, doing everything. And he did a very, very good report… It was written as one of the best reports so far” (SME4). This graduate went on to present his findings at the national level. Graduates were also credited with strengthening disease control programs through improved analytical and operational capability. In the HIV program, for example, one senior manager described a graduate’s contribution to, “forecasting and quantification for our drugs and test kits”, stating, “his learning can really help the program” (SME6). Another reflected on the broader shift in culture that graduates had helped drive: “They actually have a lot of influence in their place of work, there’s a lot of change… they are actually very, very conscious about data…. So that helps to uphold and improve indicators of programs” (SME8).

Senior managers and executives observed several challenges and noted areas for improvement. They indicated that, at times, there was underutilisation of graduates, partly due to roles that did not fully draw on their skills and partly due to a lack of confidence on the part of graduates to assert their new capabilities. One senior manager stated that graduates needed, “to put their hands up and say, hey, I’m an FETP graduate, I can go out with a team to do outbreak investigation, or I can do surveillance work” (SME6). Senior managers emphasised the need for greater program visibility and advocacy within health leadership structures: “As a program, we need to do senior management engagement, to tell them what the program’s about and how they can use this training” (SME6). Leadership development was seen as essential for enabling graduates to take on more senior roles, and formal recognition or accreditation of the qualification was highlighted as an important mechanism to strengthen career pathways. Suggestions included establishing a university partnership to award a recognised qualification and creating postgraduate or master’s pathways to extend field epidemiology training.

#### 3.5.8 Outcome and impact stories

A total of 17 stories of change were documented by graduates (Supplementary Annex B). These narratives provide examples of how the application of skills acquired through aFETPNG were translated into tangible improvements across a range of public health domains, illustrating real-world outcomes and impacts at the systems, program, and community levels.

Several examples related to the COVID-19 pandemic response. One graduate was selected by the Provincial Health Administrator to serve as the first Incident Manager for the province, applying their field epidemiology skills to analyse data, prepare reports, make evidence-based recommendations for senior management, and secure financial resources for public health interventions. As Incident Manager, they were empowered to make key decisions and were responsible for providing daily updates to the National Emergency Centre. Based on reports presented, funding was secured to build COVID-19 isolation centres and acquire a new disease surveillance vehicle for the district. Another graduate served as the COVID-19 Surveillance Cluster Lead and lead trainer for risk communication and community engagement in the province. The first COVID-19 case detected in PNG occurred while this graduate was leading the provincial surveillance cluster. Another graduate coordinated two national operational research projects to understand hesitancy of healthcare workers to swab suspected COVID-19 cases, and understand drivers of vaccine hesitancy in the general population. The results of the swabbing study were presented to the National COVID-19 Control Centre of PNG and published in a peer-reviewed journal (30), and results from the vaccine hesitancy study were presented to national and international audiences.

Other graduates shared stories of how they applied FETP skills to manage supplementary immunisation activities for measles, polio, pertussis, and rubella. These graduates achieved very high immunisation coverage and were recognised as top performers in the country. They coordinated logistics, supervised immunisation staff, and engaged with communities to address vaccine hesitancy and strengthen service delivery.

Several stories highlighted outcomes and impacts relating to disease control programs. In TB control, policy recommendations developed by a graduate during their training were implemented, including providing incentives for treatment supporters and food vouchers for multidrug-resistant TB (MDR-TB) patients. The intervention resulted in no MDR-TB patients being lost to follow-up, and a 100% treatment success rate in the province since 2020. Another graduate supervised a mass screening program for TB in correctional facilities, identifying a number of previously undetected cases. Optimisation of TB/HIV delivery was also initiated following analysis of district-level data by a graduate, who subsequently established a new integrated clinic and trained staff, improving service accessibility and reducing treatment loss-to-follow-up.

Collectively, these stories of change highlight the program’s value in building national capacity to address major public health challenges and demonstrate the breadth and depth of aFETPNG’s contributions to strengthening disease control, surveillance, outbreak response, and health systems in Papua New Guinea.

## 4 Discussion

This evaluation demonstrates that aFETPNG achieved its objectives of strengthening field epidemiology capacity, fostering an active alumni network and contributing to stronger health systems. While the COVID-19 pandemic had a major impact, the program rapidly shifted focus to supporting the national response. The COVID-19 after action review, conducted during workshop two, found that all (100%) of aFETPNG trainees were directly involved in the pandemic response, with 93% indicating that aFETPNG had prepared them well for the pandemic (10). During the response, graduates assumed leadership roles in surveillance, outbreak response, data analysis and training. Collectively, they trained over 700 health workers during the pandemic (10).

Guided by the program theory of change, this evaluation documented outputs, outcomes and impacts of aFETPNG. Contribution analysis was used to assess whether aFETPNG plausibly contributed to these outputs, outcomes, and longer-term effects, rather than seeking to attribute changes directly to the program. Contribution was assessed by examining the coherence between expected and observed changes along the theory-of-change pathway, the temporal sequencing between training, application of skills, and downstream effects, and the consistency of evidence across multiple data sources. Survey findings, in-depth interviews with graduates and senior health leaders, and documented examples of field application were triangulated to assess whether aFETPNG was a credible contributing factor to observed changes. The plausibility of alternative explanations for the changes observed was also assessed. The program theory states that providing public health workers with high-quality work-integrated field epidemiology training and mentoring would develop their confidence and competence in field epidemiology, resulting in the routine application of key field epidemiology skills in their workplaces and the strengthening of key field epidemiology functions. Over time, the cumulative effects of trainees and graduates would result in strengthened health systems and contribute to improved health outcomes at the community level. Our mixed-methods evaluation, combining survey responses with in-depth interviews of graduates and senior health leaders, allowed us to test this theory.

### 4.1 From training to competence

The training received consistently high ratings from graduates. The applied nature of the program was particularly valued, with the field projects providing structured opportunities to apply skills. The importance of the work-integrated learning model in developing field epidemiology skills has been highlighted by other evaluators (27, 29, 31). All graduates reported applying their field epidemiology skills in their current workplace. They reported a high level of confidence across a range of core field epidemiology competencies and provided examples of how they had applied their skills. Strategically recruiting aFETPNG trainees from within the public health service enabled the immediate application of skills, contributing to the link between skill development and behavioural change. While satisfaction with mentorship during workshops was high, support between workshops was rated less favourably. While this constraint did not prevent skill acquisition, it does present an opportunity for improvement.

### 4.2 From competence to behaviour change

The evaluation found extensive evidence of graduates applying skills in disease surveillance, outbreak investigation, operational research and evidence-based decision making. During the 14-month period between graduation and evaluation, graduates collectively led or supported over 50 outbreak investigations. They deployed within and across provinces, strengthening surge capacity for measles, dengue, pertussis, cholera, malaria and COVID-19 responses. Routine surveillance and data analysis capacity was also strengthened with graduates creating weekly surveillance reports and introducing digital innovations, including during COVID-19.

These findings are consistent with published literature indicating that FETPs increased the knowledge, skills, and confidence of graduates to perform key field epidemiology tasks, including disease surveillance (31–36), outbreak response (31, 32, 36–39), data analysis (32, 33), research (40), and using data for evidence-based decision making (32, 35, 36, 40, 41). Other FETPs also found that FETPs strengthened leadership skills (35) and the ability of graduates to prepare technical reports and papers (31, 33). Knowledge transfer emerged as an important outcome. All aFETPNG graduates trained or mentored colleagues, extending program benefits beyond those enrolled in the program. This *diffusion of competence* has been previously documented as an important outcome of FETPs (28, 31, 35). Graduates also disseminated findings from their field epidemiology related activities at meeting and conferences, including at national and international levels. The limited number of peer-reviewed publications highlights an opportunity to provide additional training and support to graduates to strengthen this component of the knowledge translation pathway.

### 4.3 From behaviour change to system strengthening

The theory of change indicates that the sustained application of skills by graduates will strengthen public health systems through improved surveillance, outbreak response, program quality and organisational support. We found strong evidence of progress along this pathway. Graduates worked across all levels of PNG’s health system, with 94% holding positions requiring use of their epidemiological skills. The application of skills informed disease control activities and directly contributed to mobilizing resources to sustain support for these activities. Senior managers described graduates as trusted technical advisors who “brought evidence to the table” and shifted the workplace toward a culture of evidence-based decision making. Almost all graduates indicated that their field epidemiology related work informed the development and implementation of public health programs; a majority also indicated that their work helped inform public health policy.

Several system-level improvements were attributed, in part, to the efforts of graduates. These included: enhanced outbreak detection and reporting timeliness; operational research to strengthen the national response to COVID-19; enhanced infection control measures at the facility level; improved data quality in the National Health Information System; integration of new indicators into the national surveillance system; opening of new aid posts to improve service access and delivery; and improved TB and HIV program performance. Graduates also supported the sustainability of FETPNG, serving as faculty for the Intermediate and Frontline FETP’s.

### 4.4 From system change to population benefit

The final level of the program theory anticipates improved access to health services, better program performance and ultimately healthier communities. Measuring changes in morbidity or mortality was beyond the scope and timeframe of the evaluation, however, early indicators suggest plausible progress along the impact pathway. Graduates led routine and supplementary immunisation campaigns that met or exceeded national targets. Community engagement activities were implemented to strengthen disease control programs, including malaria, TB and COVID-19. Expanded HIV/TB outreach and the establishment of new aid posts improved access to services. One graduate reduced MDR-TB treatment lost-to-follow-up and sustained a 100% treatment success rate. These examples highlight community-level benefits, consistent with the theory of change. While attribution of longer-term health impact remains complex, the direction of change aligns with the program’s theoretical pathway.

### 4.5 Contribution of other factors

There were other non-program related factors which likely contributed to the outcomes and impacts described in this evaluation. COVID-19 undeniably affected the operating environment. The pandemic created unprecedented demand for skills that graduates possessed, enhancing graduate visibility and influence, enabling immediate skill application, and accelerating progress along the change pathway. Other capacity-building initiatives, such as Rapid Response Team training, also contributed to outbreak response and system strengthening. Ongoing provincial health reforms, as well as the influence of non-governmental agencies in facilitating and strengthening disease prevention and control activities may have also contributed to the outcomes attributed to aFETPNG. While the program cannot claim sole attribution for the outcomes and impacts described, it can credibly claim to have been necessary and highly influential. Given the consistency of evidence, alignment with the theory of change, and the absence of alternative explanations to fully account for observed outcomes, the evaluation supports the conclusion that aFETPNG made a substantial contribution to strengthening epidemiological practice and health systems. While the ultimate impact of reduced morbidity and mortality requires longer-term observation, the contribution story is strong with early evidence of community level impacts of a localised nature. This evaluation provides evidence supporting the important role of FETPs in driving health system change, particularly in settings with limited epidemiological capacity.

Several recommendations have emerged from this evaluation. Strengthening mentoring, especially during field-work phases and after completion of the training, would accelerate learning and facilitate the ongoing application of skills in the workplace. Providing additional professional development opportunities following graduation would consolidate learning gains and build confidence in more advanced competencies. These opportunities could include refresher training and support for operational research, public health interventions, scientific writing and policy translation. Progress has commenced in this regard; in 2024, 14 graduates from PNGs Intermediate and Advanced level FETPs participated in professional development short courses run by the University of Newcastle and funded through Australia Awards Fellowships. These short courses were specifically developed for FETPNG graduates and focused on Excel for field epidemiologists, data management and analysis and questionnaire design (42). Formalising a graduate (alumni) network was also recommended to enhance post-graduate support. Securing funding to support collaborative projects led by graduates, including the scaling of successful interventions, would capitalise on past training investments and advance the outcomes and impacts of the program.

Ongoing advocacy with Provincial Health Authorities is needed to ensure graduates have the authority, resources and protected time required to perform core epidemiological functions. Formal recognition of field epidemiology positions within organisational structures would provide role clarity and strengthen accountability for surveillance and response. Promoting workplace cultures that value, support and recognise the professional development of graduates would further enhance the contributions of graduates. Decentralising field epidemiology training closer to the frontline was also recommended by graduates. PNG has taken significant steps in this direction with the establishment of the provincially led One Health Frontline FETP program (5, 6). This program has already trained 61 frontline workers from 4 provinces since its inception in 2022 (5).

Theory-based evaluation approaches are well suited to assessing the outcomes and impacts of FETPs (43–45). Rather than attempting to quantify impact by comparison to a counterfactual, they assemble evidence to build a credible narrative of the program’s contribution to outcomes and impacts. There were, however, several limitations associated with this evaluation. The reliance on self-reported behaviours and outcomes is vulnerable to recall and social desirability bias and the cross-sectional design prevented assessment of the long-term sustainability of outcomes and impacts. A related limitation was the lack of validation of outcomes and impacts by external sources. Some outcomes lacked specificity and quantifiable measures, and graduates were not always clear in distinguishing their own role from that of others in the change process. Future evaluations would benefit from prospectively capturing and monitoring of key outcome indicators and stories of significant change, using methods such as the Most Significant Change (46) or Outcome Harvesting (47). Although senior managers were consulted, graduate perspectives were dominant. Incorporating voices from a broader range of stakeholders, including policymakers and community beneficiaries, would strengthen the evaluation.

In conclusion, this evaluation found that aFETPNG made a substantive and credible contribution to PNG’s public health system and demonstrated measurable progress along the theory of change impact pathway. By developing PNG’s public health workforce, the program enhanced outbreak response, improved surveillance practice, strengthened program delivery and delivered benefits at the community level. These achievements were not incidental but reflected the intentional design of the program and the consistent application of skills by graduates. The evidence suggests that investments in FETPNG are advancing the long-term goal of a stronger, more resilient and more responsive public health system.

## Data Availability

The raw data supporting the conclusions of this article will be made available by the authors, without undue reservation.
