# Supplementary material for: Building epidemiological capacity to strengthen health systems: evaluating the advanced (extended) field epidemiology training program of Papua New Guinea
Source: Front Public Health. 2026 Mar 3;14:1777107. doi: 10.3389/fpubh.2026.1777107 (PMC12992315; doi:10.3389/fpubh.2026.1777107)
Supplement: Supplementary file 1 [file Supplementary_file_1.docx]

**Annex A.** Theory of Change diagram prepared during a participatory workshop, Papua New Guinea, 2019.


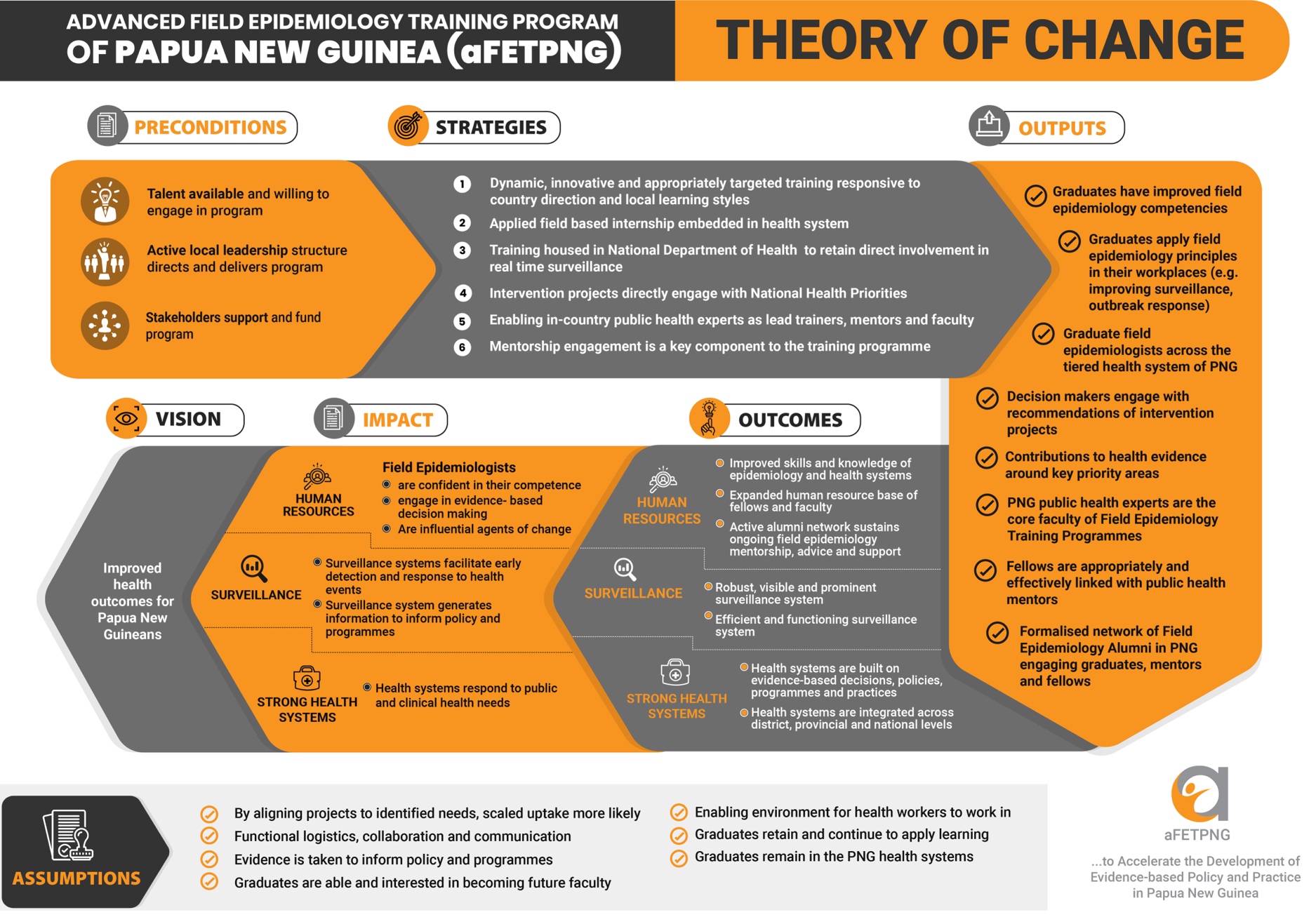


**Annex B.** Number and percentage of aFETPNG graduates who investigated outbreaks, by type of outbreak, Papua New Guinea, 2023.

| **Outbreak Type (Syndromes)^a^** | **aFETPNG Graduates**  (n=17) | |
| --- | --- | --- |
| Acute fever and rash | 10 | 59% |
| Fever with cough and sore throat (including COVID-19) | 9 | 53% |
| Acute flaccid paralysis | 9 | 53% |
| Outbreak or cluster of unexplained severe illness or death | 9 | 53% |
| Acute watery diarrhoea | 5 | 29% |
| Bloody diarrhoea | 4 | 24% |
| Prolonged fever | 3 | 18% |
| Haemorrhagic fever | 1 | 6% |
| **Outbreak Type (Specific conditions)^b^** | **aFETPNG Graduates**  (n=17) | |
| Pertussis | 4 | 24% |
| Tuberculosis | 1 | 6% |

^a^These syndromes are under surveillance in PNG’s syndromic surveillance system

^b^These specific conditions were captured in an open text field; ‘other types of outbreaks you have investigated since graduation’

**Annex C.** Examples of significant changes / outcomes / impacts achieved by aFETPNG graduates, Papua New Guinea, 2023.

| **Theme** | **Outcome/impact description and significance** | **Contribution of graduate** |
| --- | --- | --- |
| COVID-19 | I was recognised and selected by the Provincial Administrator to serve as the first Incident Manager during COVID-19 and was granted financial authority by the Provincial Administration. This was a significant outcome for me, as it allowed me to apply the new skills and knowledge gained through FETP during the COVID-19 surge. I analysed data and prepared reports with recommendations for senior management, who had confidence in my advice and allocated resources to enable interventions. | I was appointed as Incident Manager, making decisions and providing daily updates to the National Emergency Centre, while other officers submitted district updates to me on a daily basis. |
| COVID-19 | Based on my data analysis and presentation of the findings to the authorities, funding was secured to build COVID-19 isolation centres in the district. In addition, a disease surveillance vehicle was funded. This vehicle is now being used to support disease surveillance activities across the district. | I was responsible for writing up the report and presenting the findings to the authorities. |
| COVID-19 | I used my field epidemiology skills to support the COVID-19 response in my province. I was responsible for surveillance, risk communication, and community engagement. | I was the COVID-19 Surveillance Cluster Lead in my province and served as the lead trainer on risk communication and community engagement during the COVID-19 pandemic response. Notably, the first COVID-19 case detected in Papua New Guinea was identified through the surveillance system while I was leading the provincial surveillance cluster. |
| Immunisation | I was involved in organising and coordinating a supplementary immunisation activity (SIA) for polio, measles, and rubella in my district. I applied the leadership and community engagement skills I gained during FETP. The campaign ran for two months, and our team achieved a 100% coverage rate.  My district was recognised as the best performing in the province, the best performing in the Momase region, and the fourth best performing in the country. We were awarded a certificate by our CEO. Building a strong team and ensuring everyone was involved in decision-making had a huge impact, enabling us to protect our children from these diseases. | I coordinated the campaign and ensured that all six major facilities in my district received adequate support from my office, including medical supplies, per diems, drugs, and consumables. I also visited each of the six health facilities during the program to provide supervision. |
| Immunisation | I led a measles, polio, and pertussis supplementary immunisation activity at the health centre I was managing. I supervised a team of eight staff who conducted both outreach and static clinics. The team engaged with communities to address vaccine hesitancy and concerns, which were heightened following COVID-19. We achieved excellent results, reaching 100% coverage, which provided herd immunity and prevented outbreaks of these diseases. | I was the team leader at the facility level, overseeing eight staff members who assisted in implementing the program. |
| Malaria | In September this year, I assisted my colleague, the Health Information Officer, with her research project on improving data quality in the malaria program. She plans to roll out the project across the province. | I assisted my colleauge with data analysis |
| Management | One of the most important outcomes for me has been the ability to use evidence to inform decision-making. I support decisions in the various committees I represent in my workplace; they are always based on evidence from data I have collated. This represents a significant shift in practice. | My role is to provide advice and recommendations based on the evidence; then I leave it to the senior management to make the decision. |
| Management | Following aFETPNG, I was promoted from District Health Program Manager to Deputy Director of Public Health in my province. In this role, I have the opportunity to make decisions on public health matters by applying the training and skills I acquired through FETP, and I also contribute to policy decision-making. I am responsible for delivering health care services in the province and for making decisions that are locally appropriate while aligned with global public health practices. | At the management level, I provide input into decision-making and consult with colleagues and experts to gather their views. This helps ensure that decisions are evidence-informed, widely acceptable, and implementable. |
| Surveillance | I improved surveillance at the district level by strengthening processes to ensure data were validated and analysed, and that surveillance reports were prepared and shared with decision-makers and data providers. Feedback reports are provided to the district health team for health program planning, and to staff of reporting health facilities on a monthly basis.  Using surveillance data, my team was able to focus on priority public health programs, including routine immunisation. In my district, it is now routine practice that all surveillance reports are submitted to the District Disease Control/Surveillance Officer for validation, data management, and analysis before being forwarded to the Provincial Health Information Officer. This process strengthened and improved higher quality data for timely decision-making to enhance public health programs. | My contribution was establishing processes, conducting data validation, management, and analysis, as well as preparing feedback reports on the monthly performance of health indicators. These reports are produced to inform the district health team, which includes the District Health Manager, program coordinators (Family Health and Environmental Health Promotion Officers), aid post supervisors, and the Officers in Charge of the eight major reporting health facilities in the district. These officers review the program indicators and use the findings to guide the design and planning of priority health activities aimed at improving poor health indicators. |
| Surveillance | I improved the quality of data collected for the National Health Information System at the health facility level. This information is sent to the provincial health office. | I mentored and supervised health staff at the facility by reviewing the various daily data collection tools and registers with them, explaining what each variable measured, and teaching them how to collect accurate and high-quality data. |
| Training | Following graduation, I took on the role of coordinator and mentor for the FETP programs in PNG. I used my skills in mentoring, motivating, and encouraging colleagues to ensure they successfully graduated from the program and made positive impacts in their workplaces. The significance of this outcome was my contribution to building public health workforce capacity in my country, thereby supporting PNG Vision 2050. Although there are still gaps in surveillance and outbreak response capacity and capability, I am proud to be part of FETPNG and to contribute skills that help fill these gaps, equipping and enhancing our existing workforce to strengthen surveillance and respond more effectively to outbreaks and other public health emergencies. Trainees I have mentored have gone on to receive Australia Awards scholarships.  While working with FETPNG, I also applied my skills to coordinate two operational research projects during COVID-19. I coordinated and wrote up the results of a study examining the enablers and barriers experienced by healthcare workers in swabbing for COVID-19. This study was published in the International Journal of Infectious Diseases in 2021. The other study, conducted with FETP colleagues, focused on understanding the factors contributing to vaccine hesitancy in PNG. | I was responsible for coordinating FETPNG as the program convenor, where I provided mentoring and delivered training. I also co-facilitated training-of-trainer workshops for FETP faculty.  For the swabbing study, I coordinated the project and oversaw data collection and analysis, and I wrote up the final report. The FETPNG faculty supported me in designing the study.  For the vaccine hesitancy study, several FETPNG graduates and faculty were involved. I supported the study design, led the training of interviewers, and coordinated the data collection. This role was made possible by my experience with the COVID-19 swabbing study, which gave me the confidence and courage to supervise the project. I was not involved in the data analysis or report writing for the vaccine hesitancy study, but I had the opportunity to co-present the findings to international colleagues. |
| TB | There have been no losses to follow-up of MDR-TB patients in my province since 2020, following the implementation of the policy recommendations I developed during aFETPNG. My policy brief recommended incentives for treatment supporters and food vouchers for MDR-TB patients. As a result, the province has achieved zero loss to follow-up among MDR-TB patients and a 100% treatment success rate. | The project and policy brief I conducted and prepared during aFETPNG formed the basis of this intervention. I led this activity with support from Anglicare PNG. |
| TB | I investigated an increase in TB cases reported at [Provincial] prison and recommended mass screening of inmates. My recommendation was implemented using mobile X-ray and GeneXpert testing. This led to the detection of additional TB cases and the provision of treatment. | I was the TB Coordinator, providing overall supervision. |
| TB/HIV | I analysed TB and HIV data from my district and identified high rates of TB defaulters and HIV cases concentrated in one particular local-level government area. Based on this analysis, I met with the Provincial Disease Control Officer to discuss the findings and recommended that the province establish a TB and HIV clinic in this area and train staff to manage a TB/HIV program. These recommendations were approved, and the centre is now established and operating as a TB Basic Management Unit, reporting directly to the province.  As a result, all TB and HIV treatment now occurs locally rather than requiring patients to travel long distances. My research had shown that distance and the cost of transport were key barriers preventing patients from accessing treatment. The clinic opened in 2023, and its significance is clear: many TB and HIV cases are now managed at the facility, reducing loss to follow-up, lowering the risk of MDR-TB developing, and ensuring that both TB and HIV cases are properly managed. | I analysed the data and identified that most cases were coming from one particular area. I presented the results and recommended establishing a new clinic. The PDCO approved the plan and accredited the clinic as an official TB/HIV Basic Management Unit. I also provided logistical support and trained the clinic staff. |
| HIV | I was involved in improving HIV testing coverage in my province from less than 20% to 100%. This was a significant achievement for the provincial HIV program. | I coordinated the implementation of the intervention, while others were responsible for carrying it out. |
| NCDs | I reviewed the reporting pathway for non-communicable diseases and identified a gap: these conditions were not being captured in the health information system at the health department. | I helped identify a gap in the reporting of non-communicable disease data. |
