# Supplementary material for: Building epidemiological capacity to strengthen health systems: evaluating the advanced (extended) field epidemiology training program of Papua New Guinea
Source: Front Public Health. 2026 Mar 3;14:1777107. doi: 10.3389/fpubh.2026.1777107 (PMC12992315; doi:10.3389/fpubh.2026.1777107)
Supplement: Supplementary file 2 [file Data_Sheet_1.PDF]

# FETPNG Graduate Survey

## For graduates of intermediate and advanced FETPNG

## Nov 2023

**INTRODUCTION**

The PNG National Department of Health and the University of Newcastle are reviewing the Field Epidemiology Training Programs of Papua New Guinea.

As a graduate, we are interested in learning about your experiences during and following your training. The information you provide will be used to improve and inform future programs and projects. Please refer to the Participant Information Sheet for further information about this survey.

**CONSENT**

I have read the information sheet linked above. I agree to participate and give my consent. I understand that the project will be conducted as described in the Participant Information Statement, a copy of which I have read and understood. I understand that I can withdraw from the project, and I do not have to give any reason for my decision to withdraw. I understand that my personal information will remain confidential to the researchers.

☐ I consent to this survey.

**BACKGROUND**

1. What is the highest level of Field Epidemiology Training you completed in PNG?

   ☐ Intermediate FETPNG
   ☐ Advanced FETPNG

2. Did you start but not complete the advanced FETPNG?
   *(please select yes if you started the advanced FETPNG but did not complete all 3 workshops)*

   ☐ Yes
   ☐ No

3. What were the reasons for not completing the advanced FETPNG? *(check all that apply)*

- ☐ I was too busy with the COVID response
- ☐ I was too busy with other work (non-COVID)
- ☐ My manager was not supportive of me continuing with the advanced FETPNG program
- ☐ I found the training in the face-face workshops too difficult to understand
- ☐ I didn't have the necessary skills or confidence to do the field project
- ☐ I lacked mentor support to do my project (no mentoring or poor quality mentoring)
- ☐ I lacked management support to do my project
- ☐ I didn't get along with my aFETPNG mentor
- ☐ I lost interest in continuing with aFETPNG
- ☐ Field epidemiology skills no longer aligned to my job requirements
- ☐ aFETPNG was not aligned with my future career interests
- ☐ I started another training program and couldn't continue with aFETPNG
- ☐ There were personal reasons (not work related) that made it too difficult for me to continue with aFETPNG
- ☐ Other, please specify:

4. Please share any additional details regarding the reason(s) for not completing the advanced FETPNG?

5. Have you ever been a faculty member, trainer and/or mentor of FETPNG (frontline, intermediate or advanced)?

- ☐ Yes
- ☐ No

6. If YES, for what program(s) have you been a faculty, trainer and/or mentor? *(check all that apply)*

- ☐ Frontline FETPNG
- ☐ Intermediate FETPNG
- ☐ Advanced FETPNG

**LEARNING**

*NOTE: If an advanced FETPNG graduate, answer all question based on your advanced FETPNG experience, not your intermediate FETPNG experience*

7. During your FETPNG training, how would you rate:

| | **GOOD** | **AVERAGE** | **POOR** |
|---|---|---|---|
| The training content | ☐ | ☐ | ☐ |
| The training materials provided | ☐ | ☐ | ☐ |
| How the training was delivered | ☐ | ☐ | ☐ |
| The knowledge of the facilitators | ☐ | ☐ | ☐ |
| Mentoring during workshops | ☐ | ☐ | ☐ |
| Mentoring between workshops | ☐ | ☐ | ☐ |

8. Can you provide 3 suggestions on how mentoring could be strengthened for future training?

**BEHAVIOUR - KNOWLEDGE AND SKILL APPLICATION**

9. How confident would you be today conducting the following activities?

| | **VERY CONFIDENT** (I could do this without support) | **CONFIDENT** (I would need some support) | **NOT CONFIDENT** (I would need a lot of support) |
|---|---|---|---|
| Identifying unusual events from surveillance data | ☐ | ☐ | ☐ |
| Using surveillance data to guide public health programming | ☐ | ☐ | ☐ |
| Evaluating or reviewing a surveillance system and making recommendations for system improvement | ☐ | ☐ | ☐ |
| Conducting an outbreak investigation | ☐ | ☐ | ☐ |
| Summarising an outbreak investigation in a written report | ☐ | ☐ | ☐ |
| Designing and conducing an operational research project or epidemiology study | ☐ | ☐ | ☐ |
| Implementing an evidence based public health intervention | ☐ | ☐ | ☐ |
| Evaluating a public health intervention | ☐ | ☐ | ☐ |
| Creating and managing a linelist | ☐ | ☐ | ☐ |
| Conducting descriptive data analysis and interpreting results | ☐ | ☐ | ☐ |
| Giving an oral scientific presentation | ☐ | ☐ | ☐ |
| Writing an abstract for a scientific conference | ☐ | ☐ | ☐ |
| Writing a scientific manuscript for a peer-review journal | ☐ | ☐ | ☐ |
| Developing evidence-based policy recommendations from data and information | ☐ | ☐ | ☐ |
| Creating a policy brief | ☐ | ☐ | ☐ |

10. Since graduating from FETPNG, have you done the following activities?

| | **YES** | **NO** | **Don't Know** |
|---|---|---|---|
| Identified unusual events from surveillance data | ☐ | ☐ | ☐ |
| Used surveillance data to guide public health programming | ☐ | ☐ | ☐ |
| Evaluated or reviewed a surveillance system and made recommendations for system improvement | ☐ | ☐ | ☐ |
| Conducted an outbreak investigation | ☐ | ☐ | ☐ |
| Summarised an outbreak investigation in a written report | ☐ | ☐ | ☐ |
| Designed and conducted an operational research project or epidemiology study | ☐ | ☐ | ☐ |
| Implemented an evidence based public health intervention | ☐ | ☐ | ☐ |
| Evaluated a public health intervention | ☐ | ☐ | ☐ |
| Created and managed a linelist | ☐ | ☐ | ☐ |
| Conducted descriptive data analysis and interpreted results | ☐ | ☐ | ☐ |
| Given an oral scientific presentation | ☐ | ☐ | ☐ |
| Writen an abstract for a scientific conference | ☐ | ☐ | ☐ |
| Writen a scientific manuscript for a peer-review journal | ☐ | ☐ | ☐ |
| Developed evidence-based policy recommendations from data and information | ☐ | ☐ | ☐ |
| Created a policy brief | ☐ | ☐ | ☐ |

11. Is there anything that you would have liked to have learnt during your field epidemiology training that was not covered or not covered adequately?

☐ Yes
☐ No
☐ Don't know

12. Please specify what you would have liked to have learnt

13. In your current job...

| | YES | NO | Don't Know |
|---|---|---|---|
| Are you employed in a position where field epidemiology knowledge and skills are required? | ☐ | ☐ | ☐ |
| Are you provided opportunities to apply field your epidemiology skills and knowledge? | ☐ | ☐ | ☐ |
| Are you provided regular supervision? | ☐ | ☐ | ☐ |
| Do you use data to guide your decision making? | ☐ | ☐ | ☐ |
| Do you use data to help others make decisions? | ☐ | ☐ | ☐ |
| Does your work inform the development and implementation of projects or programs? | ☐ | ☐ | ☐ |
| Does your work inform the development of health policies? | ☐ | ☐ | ☐ |

14. Since graduating from FETPNG have you commenced or completed further training or professional development?

☐ Yes
☐ No
☐ Don't know

15. If YES, what type of training have you commenced or completed? *(check all that apply)*

☐ PhD
☐ Masters
☐ Certificate
☐ Other
☐ Don't know

16. Since graduating from FETPNG have you received a promotion?

☐ Yes
☐ No
☐ Don't know

17. How important was your FETPNG training in receiving this promotion?

- ☐ Very important (my promotion was directly related to my FETP training)
- ☐ Moderately important (my promotion was partly related to my FETP training)
- ☐ Not at all important (my promotion was unrelated to my training)

18. Since graduating from FETPNG have you been a member of a national, regional or international working group or committee?

- ☐ Yes
- ☐ No
- ☐ Don't know

19. If YES, please specify the name and type of each working group or committee you have been a member of

20. Since graduating from FETPNG have you transferred knowledge or skills learned in FETPNG to others through mentoring or other professional support?

- ☐ Yes
- ☐ No
- ☐ Don't know

21. Since graduating from FETPNG, please indicate where you have presented your field epidemiology related work: *(check all that apply)*

- ☐ To the general public/community
- ☐ To colleagues and/or superiors in your workplace
- ☐ At a National meeting or conference (including presentations to donors, stakeholders, partners)
- ☐ At an International meeting or conference
- ☐ I have not presented my work

**RESULTS**

22. Since graduating from FETPNG, how many outbreaks have you investigated:
*(if none, enter "0")*

As a **lead investigator** (you were in charge of coordinating the investigation) ☐

As a **support investigator** (you were part of the investigation team, but not in charge of the investigation) ☐

23. Please check all the types of outbreaks you have investigated since graduation (check all that apply)

- ☐ I have not investigated any clusters or outbreaks since graduating
- ☐ Acute flaccid paralysis
- ☐ Acute fever and rash
- ☐ Fever with cough and sore throat
- ☐ Acute watery diarrhoea
- ☐ Bloody diarrhoea
- ☐ Prolonged fever
- ☐ Haemorrhagic fever
- ☐ Outbreak or cluster of unexplained severe illness or death
- ☐ Other, please specify:

24. Have you been deployed outside of your province to support an outbreak investigation?

- ☐ Yes
- ☐ No
- ☐ Don't know

25. For any of your outbreak investigations, did you

| | YES | NO | Don't know |
|---|---|---|---|
| Lead the writing of an outbreak report | ☐ | ☐ | ☐ |
| Support the writing of an outbreak report | ☐ | ☐ | ☐ |
| Conduct a case control or cohort study | ☐ | ☐ | ☐ |

26. Since graduating from FETPNG, have you evaluated a surveillance system?

☐ Yes
☐ No
☐ Don’t know

27. If YES, What type of surveillance system(s) did you evaluate? *(check all that apply)*

☐ Event based surveillance
☐ Syndromic surveillance
☐ Disease based surveillance (e.g. TB. HIV, Malaria)
☐ Other, please specify:

28. How many surveillance systems have you evaluated since you graduated from FETPNG?

29. Since graduating from FETPNG, please indicate which of the following surveillance activities you have done: *(check all that apply)*

☐ Made an improvement to a surveillance system?
☐ Analysed data from a surveillance system
☐ Used surveillance data to detect an outbreak
☐ Used surveillance data to guide the delivery of a public health program or activity?

30. Since graduating from FETPNG, please indicate which of the following operational research activities you have done: *(check all that apply)*

- ☐ Supported an operational research project (you were part of the research team, but not the lead)
- ☐ Led an operational research project (you were the lead researcher)
- ☐ Implemented an evidence based intervention

31. Please briefly describe the operational research project(s) you have been involved in
*(skip if you have not done any operational research)*

32. Please briefly describe the intervention project(s) you have been involved with
*(skip if you have not done any intervention project)*

33. Since graduating from FETPNG, have you conducted community engagement activities:

| | YES | NO | Don't know |
|---|---|---|---|
| During the COVID-19 response? | ☐ | ☐ | ☐ |
| During a non-COVID outbreak related activity? | ☐ | ☐ | ☐ |
| For a non-outbreak related public health activity? | ☐ | ☐ | ☐ |

**OUTCOMES AND IMPACTS**

*34.* What impact has FETPNG had on you professionally? *(check all that apply)*

- ☐ I have improved data management in my workplace
- ☐ I have improved data collection in my workplace
- ☐ I have improved data analysis and interpretation in my workplace
- ☐ I have improved report writing in my workplace
- ☐ I have improved communication in my workplace
- ☐ I am collaborating with colleagues from other government sectors (e.g. environment, animal, wildlife, etc)
- ☐ I am communicating with colleagues from other government sectors (e.g. environment, animal, wildlife, etc)
- ☐ I have more confidence in my workplace
- ☐ I don’t think the training has had any impact on my professionally
- ☐ Other, please specify:

35. Please think of one of the most important outcomes or changes you have achieved or implemented since graduating from FETPNG and

(a) Describe the outcome, when and where it occurred, and who was involved?

(b) Why is the outcome significant?

(c) What was your contribution to the outcome and what was the role other people?

**DEMOGRAPHICS**

36. What is your sex?

- ☐ Male
- ☐ Female
- ☐ Other, please specify:

37. What is your age group?

- ☐ <20 years
- ☐ 20 - 29 years
- ☐ 30 - 39 years
- ☐ 40 - 49 years
- ☐ 50 - 59 years
- ☐ 60+ years

38. What is your highest education level?

- ☐ High School
- ☐ University/college certificate
- ☐ University degree or equivalent
- ☐ Post graduate diploma / certificate
- ☐ Master’s degree
- ☐ Doctorate (Medical Degree, PhD, Veterinary Medicine)
- ☐ Other, please specify:

39. What is your professional background? *(check all that apply)*

- ☐ Health Extension Officer
- ☐ Nurse
- ☐ Medical doctor
- ☐ Laboratory technician
- ☐ Animal Health Officer / Rural development officer
- ☐ Other, please specify:

40. What is your current Job Title (e.g. Surveillance Officer, Nursing Officer, Clinical HEO, PCDO, etc)?

41. How many years of experience do you have in a health-related field?

- ☐ < 1 years
- ☐ 1-5 years
- ☐ 6-10 years
- ☐ 11-15 years
- ☐ 16-20 years
- ☐ 20+ years
- ☐ Not applicable

42. What is your current employment level?

- ☐ Local level (e.g. Health Centre)
- ☐ District
- ☐ Provincial
- ☐ National
- ☐ Other, please specify:

43. What type of employer do you currently work for?

- ☐ Government
- ☐ Church
- ☐ University
- ☐ Private
- ☐ Non-government organisation (NGO)
- ☐ Other, please specify:

44. What is your province of employment?

- ☐ Central
- ☐ Simbu
- ☐ Eastern Highlands
- ☐ East New Britain
- ☐ East Sepik
- ☐ Enga
- ☐ Gulf
- ☐ Madang
- ☐ Manus
- ☐ Milne Bay
- ☐ Morobe
- ☐ New Ireland
- ☐ Oro
- ☐ Bougainville
- ☐ Southern Highlands
- ☐ Western (Fly)
- ☐ Western Highlands
- ☐ West New Britain
- ☐ Sandaun (West Sepik)
- ☐ National Capital District
- ☐ Hela
- ☐ Jiwaka
- ☐ Other, please specify:

**Thank you for completing this survey.**

**We appreciate your time and the information you have provided!**
