# Supplementary material for: Building epidemiological capacity to strengthen health systems: evaluating the advanced (extended) field epidemiology training program of Papua New Guinea
Source: Front Public Health. 2026 Mar 3;14:1777107. doi: 10.3389/fpubh.2026.1777107 (PMC12992315; doi:10.3389/fpubh.2026.1777107)
Supplement: Supplementary file 3 [file Data_Sheet_2.PDF]

Evaluating the Impact of Field Epidemiology Training in Papua New Guinea

Graduate Interview Instrument

INTERVIEWER: Please review interview guide prior to each interview

Introduction

Thank you for agreeing to participate in today's interview. The PNG National Department of Health and the University of Newcastle are reviewing the intermediate level Field Epidemiology Training Program of Papua New Guinea. We are interested in the experience of graduates and understanding better how the training has impacted you and your work.

The information you provide will help us understand the impact of the program and will be used to improve and inform future field epidemiology training programs.

As part of this project, I would like to ask you questions about some of your experiences following your graduation from iFETPNG. The interview will go for approximately 60 minutes.

You will have been sent an information sheet outlining the study, as well as a consent form.

Can I check to see if you have read the information sheet and provided your written consent?

- ☐ Yes
- ☐ No = pause interview until consent has been received

A few reminders before we start:

- Your participation is voluntary and we can stop the interview at any time
- We plan to record this interview so we can later convert it to text which allows us to accurately analyse so we can analyse themes that come from all the interviews. The audio recordings will be deleted following transcription of the audio to text.
- The information you provide me today will be combined with the information we receive from other graduates we interview; we will summarize these findings together.
- Your name will not be linked to anything you say.

Do you have any questions for me before we start?

Are you happy if I record the interview – it makes it easier as then I can focus on what you are saying instead of focussing on taking notes.

Consent to recording

- ☐ Yes
- ☐ No = pause interview until consent has been received

I am going to start recording now – *PRESS RECORD*

Questions

*Interviewer, please say the following prior to each interview: today is [say todays date] and we are in [say province name]. This is interview [say Interview ID for an audio record].

Thank you for agreeing to this interview. We appreciate the time you are sharing with us today.

1. Can you introduce yourself and your current role?

2. Have you been able to apply the knowledge or skills you learnt during your FETP to your work?

   If no

   a. What do you think are the key reasons why you haven't been able to apply your knowledge of skills? (then go to Q4)

   If yes

   a. What knowledge and skills from FETP do you most often use in your work?

   b. What knowledge and skills from FETP do you not use, or rarely use?

   c. Can you share some examples of how you have applied your FETP knowledge or skills in you work?

      Interviewer: following the response, please prompt for specific examples relating to key the following FETP competencies if necessary

      [Prompts] Have you had the opportunity to collect, analysis and/or interpret data? Can you tell me more about that?

      - Was that data used to make decisions?
      - Were there any outcomes or impacts resulting from your data collection, analysis and/or interpretation work? Can you tell me about these changes/outcomes and impacts? Are these changes/outcomes/impacts ongoing?

      [Prompts] Have you had the opportunity to work in <u>surveillance</u> since graduation? Can you tell me more about that?

      - Were there any outcomes or impacts from your work in surveillance? Can you tell me about these changes/outcomes and impacts? Are these changes/outcomes/impacts ongoing?

      [Prompts] Have you had the opportunity to participate in any <u>outbreak investigations</u> since graduating? Can you tell me more about that?

      - What was your role during the outbreak investigation?
      - Were there any outcomes or impacts from your outbreak investigations? Can you tell me about these changes/outcomes and impacts? Are these changes/outcomes/impacts ongoing?

[Prompts] Have you had the opportunity to conduct any <u>operational research</u> since graduation? Can you tell me more about that?

- What was your role in this project? How did your FETP training help you in this project?
- Were there any outcomes or impacts from your operational research? Can you tell me about these changes/outcomes and impacts? Are these changes/outcomes/impacts ongoing?

[Prompts] Have you had the opportunity to use your FETP knowledge and skills to make any <u>changes in your workplace</u>? These changes could be things like changes to work processes, practices, or programs?

- What was your role in this change?
- Were there any outcomes or impacts from these changes? Can you tell me about these changes/outcomes and impacts? Are these changes/outcomes/impacts ongoing?

3. What were some of the key factors which enabled you to apply your knowledge and skills in your work?

   [Prompts] Can you tell me more? Can you clarify what you mean by […]? Were there any other factors?

4. Is there anything that would help you apply more of your FETP knowledge and skills, or apply them more effectively?

   [Prompts] Can you tell me more? Can you clarify what you mean by […]? Were there any other factors?

5. Can you share any example of how the training had an unexpected positive impact on you personally, or on your work?

   [Prompts] Can you tell me more? Can you clarify what you mean by […]? Were there any other factors?

6. Can you share any examples of how the training had an unexpected negative impact on you personally, or on your work?

   [Prompts] Can you tell me more? Can you clarify what you mean by […]? Were there any other factors?

7. Can you describe a single significant change that occurred as a result of your field epidemiology training?

   [When I refer to a significant change, I mean something you did following your training that resulted in a change in an activity, action, a behaviour or practices of an individual, group or organization]

   [Prompts] (Interviewer note: avoid moving through these prompts like a shopping list).
   - Can you tell me more about who was involved?
   - Can you tell me more about what changed?
   - Can you tell me about when and where this change occurred?
   - Can you share more about what your contribution was to this change? Were others also involved; what was their contribution?
   - Can you tell me more about the significance of this change? How did it make a difference?

8. Do you have any recommendations for the FETPNG program?

9. Is there anything further you would like to share regarding your FETP training or the impact it has had on you or your work?

Thank you for your time and input. [STOP RECORDING]

Evaluating the Impact of Field Epidemiology Training in Papua New Guinea

Manager Interview Instrument

INTERVIEWER: Please review interview guide prior to each interview

Introduction

Thank you for agreeing to participate in today's interview.

The PNG National Department of Health and the University of Newcastle are reviewing the Field Epidemiology Training Programs of Papua New Guinea (FETPNG). We are interested in your experience managing graduates for FETPNG.

The information you provide will help us understand the impact of the program and will be used to improve and inform future field epidemiology training programs.

As part of this review, we are asking managers of FETP graduates about the graduates and the program. The interview will go for approximately 30-45 minutes.

You will have been sent an information sheet outlining the study, as well as a consent form. Have you read the information sheet and provided your written consent:

- ☐ Yes
- ☐ No = pause interview until consent has been received

A few reminders before we start:

- Your participation is voluntary and we can stop the interview at any time
- We plan to record this interview so we can later convert it to text which allows us to accurately analyse so we can analyse themes that come from all the interviews. The audio recordings will be deleted following transcription of the audio to text.
- The information you provide me today will be combined with the information we receive from other graduates we interview; we will summarize these findings together.
- Your name will not be linked to anything you say.

Do you have any questions for me before we start?

Are you happy if I record the interview – it makes it easier as then I can focus on what you are saying instead of focussing on taking notes.

Consent to recording

- ☐ Yes
- ☐ No = pause interview until consent has been received

I am going to start recording now – *PRESS RECORD*

Questions
*Interviewer, please say the following prior to each interview: today is [say todays date] and we are in [say province name]. This is interview [say Interview ID for an audio record].

Interviewer note; please ask about each graduate they line-manage if >1. Do as separate interviews.

1. Can you please introduce yourself and your current role with [name of employer]?

2. What is your relationship to [FETP graduates(s)]? What role do they hold in your organization?

   [Prompts]
   - How long have you managed [graduate(s)]?
   - Were you their manager before they started FEPTNG?
   - Does [graduate(s)] manage anyone? How many people do they manage?

3. Have you noticed any changes in [graduate's] work since they completed their field epidemiology training?

   [Prompts]
   - Can you tell me more about that?
   - Has they been able to give them more responsibilities since they competed their training?
     - [if yes] Can you tell me more the new responsibilities they were given
     - [if yes] Were these new responsibilities related to the new knowledge and skills gained from their field epidemiology training?
   - Are there any other changes you have noticed in [graduate]?
   - Are there any other changes you have noticed in their work?

4. What do you think are some of the most valuable skills [graduate(s)] has applied from their field epidemiology training? When we refer to field epidemiology, we are referring to a range of skills including disease surveillance, data analysis, outbreak response, operational research, evidence based-decision making.

   [Prompts]
   - Can you tell me more about that? Why are these skills valuable?
   - Are there any other field epidemiology related skills they have applied in their work?

5. Are there any barriers which have limited [graduate(s)] ability to use their field epidemiology skills in the workplace?

   [Prompt]
   - Is there anything that would support [graduate] apply more of their field epidemiology skills in their work?

6. Have [graduate(s)] shared their field epidemiology knowledge and skills through training or mentoring of others?

   [Prompt]
   - If yes, approximately how many trainings / individuals trained by [graduate(s)]

7. Are there areas where [graduate(s)] would benefit from additional training?

   [Prompt]
   - Are there any other specific areas of training relating to field epidemiology?

8. Can you share some examples of how [graduate(s)] contributed to public health activities in their workplace following their graduation?

   [Prompts]
   - Has [graduate] contributed to data collection, analysis and/or interpretation related activities?
     - Can you tell us more about that?
     - Was the data used to make any decisions? What decisions were made and who made those decisions?
     - Was there any outcomes or impacts associated with these activities? Can you tell us these outcomes and impacts? Are they ongoing?

   - Has [graduate] contributed to any disease surveillance activities?
     - What was/is their role in disease surveillance?
     - Did they contribute to any changes to disease surveillance? How did these changes affect surveillance? Have these changes been sustained?

   - Has [graduate] contributed to the identification and response to disease outbreaks?
     - What type of outbreak/s) have they identified and/or responded to?
     - What was their role on the outbreak investigation team?
     - Have there been any changes in the way outbreaks have been identified and/or responded to since [graduate(s)] completed FETPNG? Can you tell us about those changes? Have the changes been sustained?

   - Has [graduate] conducted any operational research since their graduation?
     - Can you tell us about that operational research?
     - What were the outcomes or impacts of this project?
     - Were any interventions put in place based on the findings?

   - Has [graduate] contributed to changing a workplace process or practice that you are aware of?
     - Can you tell us about that change in process or practice?
     - What has the results of that change?

   - Has [graduate] contributed to changing a health program or policy?
     - Can you tell us about that change? [probe for both health program and policy]
     - Did the change result in any change in key performance indicators?
     - What was the outcome or impact of the change(s) made?

9. Is there anything further you would like to share regarding FETP training, its graduates and the impact it has had in the workplace?

Thank you for your time and input. [STOP RECORDING]

[Interviewer: as appropriate, please seek input on the graduates outcome statement(s) after you conclude the interview]

Evaluating the Impact of Field Epidemiology Training in Papua New Guinea

Senion Executive Interview Instrument

INTERVIEWER: Please review interview guide prior to each interview

Introduction

Thank you for agreeing to participate in today's interview.

The PNG National Department of Health and the University of Newcastle are reviewing the Field Epidemiology Training Programs of Papua New Guinea (FETPNG). We are interested in your experience managing graduates for FETPNG.

The information you provide will help us understand the impact of the program and will be used to improve and inform future field epidemiology training programs.

As part of this review, we are asking managers of FETP graduates about the graduates and the program. The interview will go for approximately 30-45 minutes.

You will have been sent an information sheet outlining the study, as well as a consent form. Have you read the information sheet and provided your written consent:

- ☐ Yes
- ☐ No = pause interview until consent has been received

A few reminders before we start:

- Your participation is voluntary and we can stop the interview at any time
- We plan to record this interview so we can later convert it to text which allows us to accurately analyse so we can analyse themes that come from all the interviews. The audio recordings will be deleted following transcription of the audio to text.
- The information you provide me today will be combined with the information we receive from other graduates we interview; we will summarize these findings together.
- Your name will not be linked to anything you say.

Do you have any questions for me before we start?

Are you happy if I record the interview – it makes it easier as then I can focus on what you are saying instead of focussing on taking notes.

Consent to recording

- ☐ Yes
- ☐ No = pause interview until consent has been received

I am going to start recording now – *PRESS RECORD*

Questions

*Interviewer, please say the following prior to each interview: today is [say todays date] and we are in [say province name]. This is interview [say Interview ID for an audio record].

1. Can you please introduce yourself and your current role with [name of employer]?

2. What has been your involvement with FEPTNG or graduates of FETPNG?

   [Prompt]
   - How long have you been involved with FETPNG?

3. Are you aware of how the FETP graduates are being utilised in their workplaces?

   [Prompts]
   - How well do you feel graduates are being utilised in the workplace?
   - Are there any barriers which may limit the application of field epidemiology skills in the workplace?
   - What could be done to improve how graduates are utilised in the workplace?

4. From your perspective, how has the capacity and capability of field epidemiology changed over time in PNG? When we refer to field epidemiology, we are referring to a range of skills including disease surveillance, data analysis, outbreak response, operational research, evidence based-decision making.

   [Prompts]
   - What role has FETPNG played in this change?
   - What specific field epidemiology skills do you feel are most valuable at this given time in PNG?

5. Do you have any examples how FETP graduates have applied their knowledge and skills to contributed to public health practice in PNG?

   [Prompts]

   - Do you have any example of how <u>surveillance</u> has been changed due to the input of field epidemiology graduates?
   - Do you have any example of how <u>outbreak response</u> has been changed due to the input of field epidemiology graduates?
   - Do you have any example of how <u>public health program delivery</u> has been changed due to the input of field epidemiology graduates?

6. Have you used data or evidence generated by field epidemiology graduates to support for decision making?

   [Prompts]

   - Can you tell me more about that?
   - How did this data/evidence help? What was your decision? What was the outcome/impact of this decision?

7. What is your vision for FETPNG going forward?

   [Prompts]

   - How can FEPTNG increase its impact on the health systems of PNG
   - Do you have any other recommendations for FETPNG?

8. Is there anything further you would like to share regarding your FETP training, it’s graduates or the impact the program is having on PNG’s health system?

Thank you for your time and input. [STOP RECORDING]
